# Ethnicity-specific association between *TERT* rs2736100 (A > C) polymorphism and lung cancer risk: a comprehensive meta-analysis

**DOI:** 10.1038/s41598-023-40504-y

**Published:** 2023-08-15

**Authors:** Xiaozheng Wu, Gao Huang, Wen Li, Yunzhi Chen

**Affiliations:** Department of Preclinical Medicine, Guizhou University of Traditional Chinese Medicine, Guiyang, 510025 China

**Keywords:** Cancer genetics, Lung cancer, Oncogenes, Cancer genetics, Oncogenes

## Abstract

The rs2736100 (A > C) polymorphism of the second intron of Telomerase reverse transcriptase (*TERT*) has been confirmed to be closely associated with the risk of Lung cancer (LC), but there is still no unified conclusion on the results of its association with LC. This study included Genome-wide association studies (GWAS) and case–control studies reported so far on this association between *TERT* rs2736100 polymorphism and LC to clarify such a correlation with LC and the differences in it between different ethnicities and different types of LC. Relevant literatures published before May 7, 2022 on ‘*TERT* rs2736100 polymorphism and LC susceptibility’ in PubMed, EMbase, CENTRAL, MEDLINE databases were searched through the Internet, and data were extracted. Statistical analysis of data was performed in Revman5.3 software, including drawing forest diagrams, drawing funnel diagrams and so on. Sensitivity and publication bias analysis were performed in Stata 12.0 software. The C allele of *TERT* rs2736100 was associated with the risk of LC (Overall population: [OR] = 1.21, 95%CI [1.17, 1.25]; Caucasians: [OR] = 1.11, 95%CI [1.06, 1.17]; Asians: [OR] = 1.26, 95%CI [1.21, 1.30]), and Asians had a higher risk of LC than Caucasians (C vs. A: Caucasians: [OR] = 1.11 /Asians: [OR]) = 1.26). The other gene models also showed similar results. The results of stratified analysis of LC patients showed that the C allele was associated with the risk of Non-small-cell lung carcinoma (NSCLC) and Lung adenocarcinoma (LUAD), and the risk of NSCLC and LUAD in Asians was higher than that in Caucasians. The C allele was associated with the risk of Lung squamous cell carcinoma (LUSC) and Small cell lung carcinoma(SCLC) in Asians but not in Caucasians. NSCLC patients ([OR] = 1.27) had a stronger correlation than SCLC patients ([OR] = 1.03), and LUAD patients ([OR] = 1.32) had a stronger correlation than LUSC patients ([OR] = 1.09).In addition, the C allele of *TERT* rs2736100 was associated with the risk of LC, NSCLC and LUAD in both smoking groups and non-smoking groups, and the risk of LC in non-smokers of different ethnic groups was higher than that in smokers. In the Asians, non-smoking women were more at risk of developing LUAD. The C allele of *TERT* rs2736100 is a risk factor for LC, NSCLC, and LUAD in different ethnic groups, and the Asian population is at a more common risk. The C allele is a risk factor for LUSC and SCLC in Asians but not in Caucasians. And smoking is not the most critical factor that causes variation in *TERT* rs2736100 to increase the risk of most LC (NSCLC, LUAD). Therefore, LC is a multi-etiological disease caused by a combination of genetic, environmental and lifestyle factors.

## Introduction

Lung cancer (LC) is one of the cancers with a high mortality rate in the world, accounting for approximately one quarter of all cancer deaths^1^. And smoking is currently considered to be a major risk factor for it^2^. In addition, exposure to environmental factors such as radon, secondhand smoke and dust, asbestos, cooking fumes and air pollution are also the main causes of LC in non-smokers^3–6^. However, it’s not only the environmental factors but also genetic differences that contribute to LC susceptibility. Over the past two decades, multi-population Genome-wide association studies(GWAS) have identified dozens of risk loci for LC^7,8^, and most of these loci are concentrated in 5p15.33 (Telomerase reverse transcriptase—Cleft lip and cleft palate transmembrane protein 1)*TERT-CLPTM1L* region^9–12^. Several precise localization studies in the following years have also identified some new LC risk loci in this region^13–15^. Telomeres are consisted of repeated "TTAGGG" at the ends of chromosomes that gradually shorten in length with each round of cell division until cell cycle arrest is triggered, of which process is known as replicative senescence^16–19^. Telomeres can normally be elongated by the ribonucleoprotein telomerase to maintain the replication potential^20,21^. In human cancer cells, however, telomerase has been activated to escape the initial growth arrest and continue to divide^22^. Unlimited cell growth and proliferation following the activation of telomerase is one of the clinical cancer phenotypes^23–25^. It has been proved that long telomeres can promote the survival of cells with acquired oncogenic DNA alterations, thereby promoting tumorigenesis^26–28^. Telomerase is consisted of a catalytic protein component encoded by the *TERT* gene and an RNA template encoded by the Telomerase RNA component(*TERC)*. Among them, *TERT* is located at the short arm 15.33 of chromosome 5 (5p15.33), which is responsible for encoding the catalytic subunit of telomerase^29^, regulating the expression level of telomerase, and maintaining telomere length, chromosomal stability and cell proliferation by adding "TTAGG" repeats at the end of the chromosomes^30,31^.

Variations of the *TERT* promoter are an important prerequisite for high telomerase expression to stabilize telomere length^32^, and this process has been observed in cancer cells^23^. Polymorphic genes in *TERT* and *TERC* have been reported to be associated with telomere length^33–35^, and longer telomere length contributes to increasing the risk of LC, especially for Lung adenocarcinoma (LUAD)^36–38^. In addition, the *TERT* gene is significantly overexpressed in LC tissues, which may also confirm the underlying mechanism of LC risk^39^. However, the association between LC risk and telomere length is inconclusive as telomere length varies with the histological type of LC^40,41^. Several single nucleotide polymorphisms (SNPs) in the *TERT* locus have been reported to be associated with cancer risk, and these SNPs are located in the exons or introns of *TERT* or its promoter^42^. The rs2736100 (A > C) polymorphism located in the second intron of *TERT* is the most common SNP in the *TERT* gene, and its association with cancer susceptibility, including LC, has been reported in various malignant tumors^29^. In *TERT* rs2736100, the C allele upregulates *TERT* expression in normal and LC tissues^19^ and is associated with longer telomere length^35,43^. Studies also have found that the increased telomere length of the C allele is associated with cancer^44^. Some studies have also shown an increased frequency of the C allele of *TERT* rs2736100 in LC patients^9,45–48^. These evidences imply that the C allele upregulates *TERT* expression, maintains and prolongs telomere length, and thus increases the risk of LC. In addition, some studies have conducted racial stratification analysis for different types of LC and proved that the influence of *TERT* variants in Asians is stronger than that in Caucasians^45,49^. These results in turn imply that the frequency of *TERT* rs2736100 variants varies across ethnic populations. However, there are some studies have not found the association between the C allele and LC^50,51^. The reasons for these different results may also be related to different ethnicities, countries, research methods, sample sizes, LC types, and linkage disequilibrium patterns. Therefore, there’s inconsistency in the results of the association of *TERT* rs2736100 with LC. While meta-analysis is an effective way to combine data to increase the sample size, obtain sufficient power to clarify inconsistent results in genetic association studies^52^.

Several meta-analyses have reported the association of the *TERT* rs2736100 polymorphism with LC, but these meta-analyses have some shortcomings: some meta-analyses have shown an increased frequency of the C allele of *TERT* rs2736100 in LC patients but they ignored the effect of different ethnic groups^53,54^; there are some meta-analyses of ethnic stratification of rs2736100, but most of them focused on different types of cancer, and they were not subjected to a stratified analysis of LC^48^; some studies have done racially stratified meta-analyses for different types of LC, however, they are outdated^55^. Therefore, there is still a lack of a unified conclusion on the association of *TERT* rs2736100 polymorphism with LC, especially the variability of this association in different ethnic populations and in different LC subtypes. This study included data from GWAS and case–control studies reporting the association of *TERT* rs2736100 (A > C) polymorphisms with LC up to date with the aim of clarifying its association with LC and the differences in this association between different ethnicities and different types of LC.

## Data and methods

### Inclusion and exclusion criteria

#### Inclusion criteria

① They must be GWAS or case–control studies on *TERT* rs2736100 A/C gene polymorphism and LC susceptibility, the language should be English, and the detection methods and means should be accurately described; ② The gene frequency data can be used to calculate the Odds ratio(OR) and 95% Confidence interval(95% CI); ③ The distribution of genotype frequency of all controls conforms to Hardy–Weinberg(HWE)^56^; ④ The score of Newcastle Ottawa scale(NOS)^57^ should be no less than 7 (≥ 7).

#### Exclusion criteria

① Studies without allele-related data; ② Studies of the types of reviews, meta-analyses, conference reports and case reports; ③ Studies with pedigree as the reporting object; ④ same studies have published for multiple times, only the one with the most complete data will be included, and the others will be excluded.

### Outcomes

The pre-specified primary outcomes were to investigate whether *TERT* rs2736100 A/C polymorphism increased the risk of LC in the overall population. The secondary outcomes were to determine whether there were differences in the intensity of the association between the *TERT* rs2736100 A/C polymorphism and LC (including various subtypes) between different ethnic groups.

### Retrieval strategy

Relevant literatures on *TERT* rs2736100 polymorphism and LC susceptibility in PubMed, EMbase, CENTRAL, MEDLINE databases published before May 7, 2022 were searched by theme words and keywords. The language was limited to English.

Search terms in PubMed(Table 1/Table S1 in supplemental content): "Lung cancer" OR "LC"AND "rs2736100" OR "*TERT*" AND "polymorphism". Manual retrieval and literature tracing methods were used at the same time to expand the search scope.

**Table 1:** PubMed search strategy.

Number	Search terms
#1	Mesh descriptor: (Lung cancer) explode all trees
#2	(LC [Title/Abstract]) OR (Lung cancer [Title/Abstract])
#3	OR 1–2
#4	Mesh descriptor: (Telomerase reverse transcriptase) explode all trees
#5	(TERT [Title/Abstract]) OR Telomerase reverse transcriptase [Title/Abstract])OR rs2736100 [Title/Abstract])
#6	OR 4–5
#7	Mesh descriptor: (polymorphism) explode all trees
#8	3 AND 6 AND 7

### Literature screening and data extraction

Two relatively independent researchers (X–ZW and WL) completed literature searching and screening according to the inclusion criteria. They cross checked and discussed them afterwards. For the literatures with different opinions, the third party (Y–ZC) made the decision. For some literatures with incomplete data, they tried to contact the author by e-mail to obtain the complete data. Finally, data extraction was carried out for the literatures being chosen after the final decision. These data include: author, year of publication, country, ethnicity, smoking status of subjects, type of LC, number of cases in case and control groups, frequency of each genotype in case and control groups, and the OR and 95% CI of each genotype.

### Literature quality evaluation

The quality of the included literature was evaluated in the NOS^57^ (X–ZW and WL), and those with a score of no less than 7 were considered as literatures with high-quality.

### Statistical methods

The HWE of the genotypes of the controls was detected by Pearson's chi-square test in SPSS 22.0 software. All results were statistically counted and analyzed in Revman 5.3 software, including drawing forest plots and funnel plots. When there was no heterogeneity among all studies or among all subgroups (*P* > 0.1 or I^2^< 50%), the fixed-effects model was used for statistical analysis; otherwise, the random-effects model was used for statistical analysis. The effect size and effect value of the statistical results were presented by OR value and 95% CI. Begg's Test and Egger's Test were performed in Stata 14.0 software to assess publication bias among studies, and sensitivity analysis was performed to assess the results of statistical analysis with greater heterogeneity. TSA 0.9.5.10 software was performed for the Trial sequential analysis(TSA) tests to evaluate the stability of the conclusion ((Type I error) probability = 5%, statistical test power = 80%, relative risk reduction = 20%).

### Ethics and dissemination

This review does not require ethical approval because the included studies are published data and do not involve the patients’ privacy. The results of this review will be reported in accordance with the PRISMA extension statement and disseminated to a peer-reviewed journal.

## Results

### Characteristic of eligible studies

**Figure 1:**
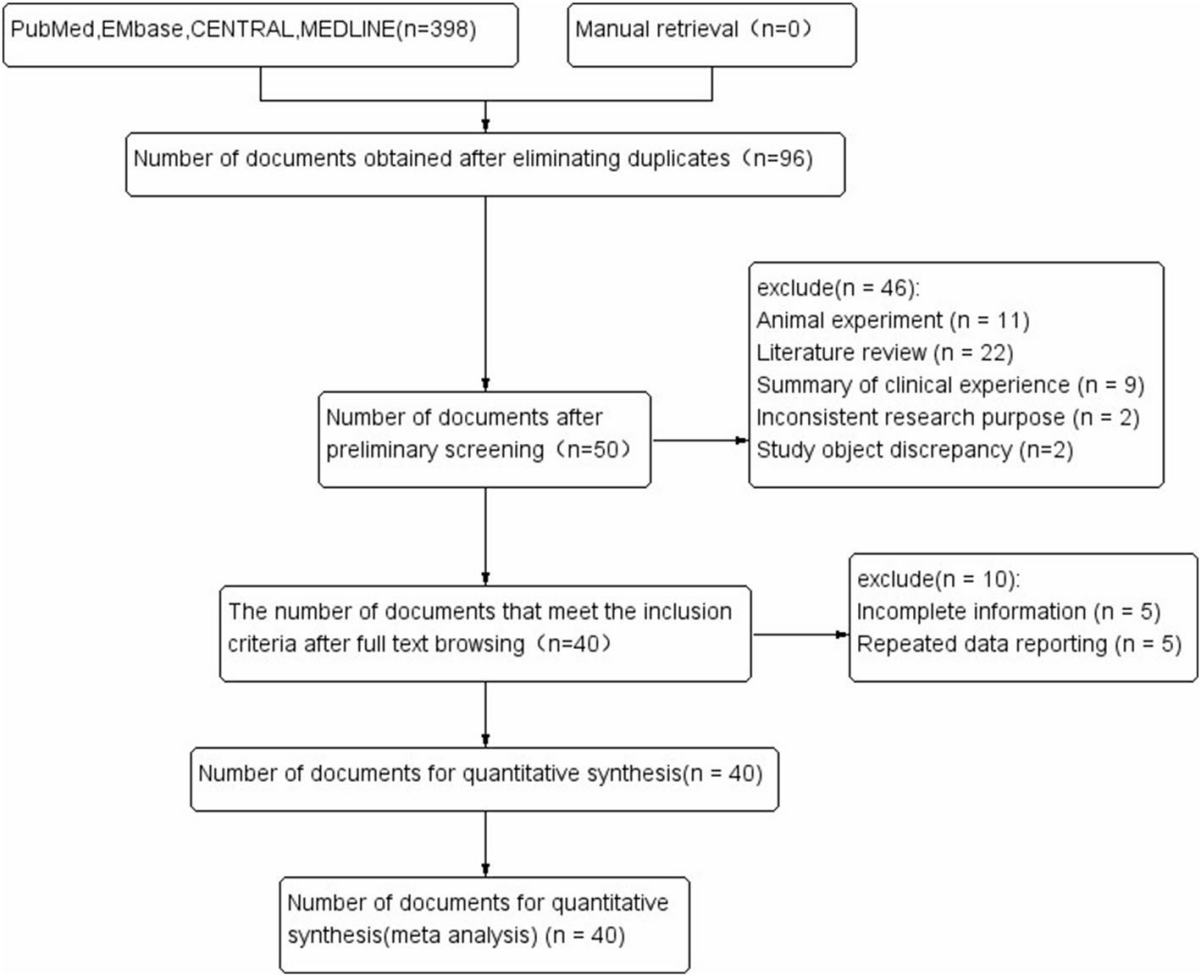
PRISMA literature screening flow chart.

A total of 398 literatures were initially retrieved from the 4 databases and 43 studies in 40 literatures were finally included after the screening^10,12,14,39,45–47,50,51,58–88^ , of which there were 25 GWAS in 22 literatures^10,12,14,45,47,50,58–61,63–65,68,69,74,76,77,79,80,87,88^. And a flow chart was made according to the PRISMA statement (Fig. 1). Among these studies, 12 in Caucasians and 31 in Asians were included. There were 99,941 LC patients (including 36,943 Caucasian patients and 62,998 Asian patients) and 131,856 controls (Tables 2, 3). All 43 studies had high NOS^57^ assessment scores (≥ 7), indicating that they are all at low risk of bias (Table 4).

**Table 2:** Basic features of the included study (1).

ID	Studies	Year	Country	Ethnicity	Type of LC	LC(n)	Controls(n)	Gender (male %)		Age (years)		Percentage of smokers (%)	
								LC	Controls	LC	Controls	LC	Controls
1	Bae^58^	2012	South Korea	Asian	LC	1094	1100	76.51%	76.36%	60.7 ± 9.3	60.6 ± 9.3	79.10%	66.40%
2	Brenner (Phase 1)^59^	2013	Europe, North America	Caucasian	LC	4441	5194	–	–	–	–	Partial smoking	Partial smoking
3	Brenner (Phase 2)^59^	2013	USA	Caucasian	LC	5699	5818	–	–	–	–	Partial smoking	Partial smoking
4	Broderick (Phase 1)^60^	2009	UK	Caucasian	LC	1952	1438	59.76%	–	57 ± 6	–	Partial smoking	Partial smoking
5	Broderick (Phase 2)^60^	2009	UK	Caucasian	LC	2465	3005	68.04%	49.31%	72 ± 7	61 ± 11	Partial smoking	Partial smoking
6	Chen^50^	2012	China	Asian	LC	196	229	77.55%	73.36%	55.9 ± 10.3	54.6 ± 10.2	62.76%	48.03%
					LUAD	96	229						
					LUSC	44	229						
					SCLC	16	229						
7	Cheng^61^	2016	China	Asian	LC	2331	3077	73.40%	67.79%	52.34%(≥ 60 )	53.56%(≥ 60)	64.05%	42.96%
8	Dong^14^	2017	China	Asian	NSCLC	192	278	72.90%	71.20%	46.90%(≥ 60 )	48.20%(≥ 60)	67.20%	47.50%
9	Furuie^62^	2021	Japan	Asian	LC	462	379	62.10%	74.70%	68 (62–73)	58 (48–65)	66.90%	44.80%
10	Hosgood^12^	2015	Asia	Asian	LC	1730	1349	0%	0%	52.30%(≥ 59 )	52.8%(≥ 59)	Non-smoking	Non-smoking
11	Hsiung^45^	2010	Asia	Asian	LC	2308	2321	0%	0%	56.3–63.4	56.3–64.7	Non-smoking	Non-smoking
					LUAD	1748	2321						
					LUSC	177	2321						
12	Hu^10^	2011	China	Asian	LC	8559	9378	69.05%	66.77%	59.11–60.08	56.51–62.45	58.36%	39.98%
					LUSC	3017	9378						
					LUAD	4323	9378						
					SCLC	780	9378						
					LC Smoker	5026	3815						
					LC Non smoker	3533	5563						
13	Ito^63^	2012	Japan	Asian	LC	716	716	74.16%	74.16%	–	–	75.20%	59.36%
14	Jaworowska^64^	2011	Poland	Caucasian	LC	855	844	73.70%	73.70%	61 (28–88)	61 (28–88)	87.50%	49.90%
15	Jin^65^	2009	China	Asian	NSCLC	1212	1339	74.40%	74.70%	48.50%(> 60)	48.10%(> 60)	64.40%	44.50%
					LUAD	711	1339						
					LUSC	374	1339						
					NSCLC Smoker	786	598						
					NSCLC Non smoker	425	746						
16	Kohno^66^	2011	Japan	Asian	LUSC	370	320	90.19%	56.92%	62.7 ± 7.6	62.5 ± 11.3	97%	45%
17	Lan^67^	2013	China	Asian	LC	193	197	0%	0%	58.14%(≥ 60 )	59.07%(≥ 60 )	7.44%	4.65%
18	Lan^68^	2012	Asia	Asian	LC	5505	4543	0%	0%	58.8 ± 11.2	55.1 ± 13.7	Non-smoking	Non-smoking
19	Landi^69^	2009	USA, Europe	Caucasian	LC	5739	5848	–	–	–	–	93.69%	76.03%
					LUAD	1730	5848						
					LUSC	1400	5848						
					SCLC	678	5848						
					LC Smoker	5356	4425						
					LC Non smoker	362	1402						
20	Li^70^	2012	China	Asian	LC	2283	2785	73.72%	73.21%	60.09 ± 10.29	60.56 ± 9.58	64.91%	45.53%
21	Li^51^	2016	China	Asian	LC	391	337	67.52%	67.66%	58.63 ± 8.8	38.8 ± 10.7	No description	No description
22	Liu^71^	2015	China	Asian	LC	288	317	48.36%	49.22%	59.63 ± 10.82	43.06 ± 15.02	No description	No description
23	Machiela^72^	2015	Asia	Asian	LC	5457	4493	0%	0%	63.00%(≥ 50)	63.00%(≥ 50)	Non-smoking	Non-smoking
24	Mandour^73^	2020	Egypt	Caucasian	LC	40	40	50%	22.50%	44.13 ± 16.18	34.45 ± 9.98	Non-smoking	Non-smoking
					NSCLC	36	40						
					SCLC	2	40						
					LUAD	26	40						
					LUSC	4	40						
25	McKay^74^	2008	USA, Europe	Caucasian	LC	2971	3746	–	–	–	–	Partial smoking	Partial smoking
26	Miki^75^	2010	Japan, South Korea	Asian	LUAD	2086	11,034	53.35%	68.42%	64.8–58.9	50.5–58.9	49.10%	59.47%
27	Myneni^46^	2013	China	Asian	LC	352	447	50.60%	50.20%	61.10%(≥ 55)	52.40%(≥ 55)	55.10%	38.80%
28	Pande^76^	2011	USA	Caucasian	LC	1681	1235	59.50%	40.50%	63.5 ± 11	57.2 ± 13.2	72.52%	58.87%
29	Seow^77^	2017	Asia	Asian	LUAD	7505	7070	0%	0%	57.9–64.6	44.2–62.0	Non-smoking	Non-smoking
30	Shiraishi^78^	2016	Japan	Asian	LUAD	6830	15,155	52.29%	56.30%	64.1	47.7	54.36%	50%
31	Shiraishi^79^	2012	Japan	Asian	LUAD	4648	12,364	46.92%	56.54%	58.8–63.3	44.5–56.6	48.71%	48.63%
					LUAD smoker	2269	6012						
					LUAD Non smoker	2368	5182						
32	Truong^80^	2010	North America, Asia	Asian	LC	1686	2101	50.00%	42.00%	87.00%(≥ 50)	77.00%(≥ 50)	59.62%	37.53%
					LC Smoker	982	759						
					LC Non smoker	671	1264						
33	Truong^80^	2010	USA, Europe	Caucasian	LC	9126	11,812	58.00%	57.00%	89.00%(≥ 50)	89.00%(≥ 50)	89.47%	63.29%
					LC Smoker	8008	6855						
					LC Non smoker	934	3972						
34	Wang^81^	2014	China	Asian	NSCLC	1552	1605	60.89%	58.44%	55.6 (29–82)	52.3 (21–29)	73.20%	53.80%
					LUAD	746	1605						
					LUSC	596	1605						
35	Wang^82^	2016	China	Asian	LC	500	500	61.00%	60.40%	84.00%(≥ 50)	71.90%(≥ 50)	Partial smoking	Partial smoking
36	Wang^47^	2010	UK	Caucasian	LC	239	553	57.74%	18.99%	67 (26–87)	63 (21–91)	Non-smoking	Non-smoking
					SCLC	39	553						
					NSCLC	200	553						
					LUAD	112	553						
					LUSC	48	553						
37	Wei^39^	2015	China	Asian	NSCLC	702	2520	64.29%	34.68%	56.7–58.7	60.5 ± 10.3	50.14%	19.68%
38	Xing^83^	2016	China	Asian	NSCLC	418	410	65.80%	61.20%	70.8 ± 16.7	71.9 ± 16.1	53.90%	49.80%
39	Yang^84^	2010	USA	Caucasian	LC	1735	1036	51.47%	40.25%	64.4 ± 10.3	64.5 ± 10.8	81.04%	39.77%
					LC Smoker	1406	412						
					LC Non smoker	329	624						
40	Yin^85^	2014	China	Asian	LC	524	524	0%	0%	56.1 ± 11.9	56.8 ± 11.1	Non-smoking	Non-smoking
					LUAD	365	524						
41	Yoo^86^	2020	South Korea	Asian	LC	699	606	100%	100%	61.1 ± 8.0	60.6 ± 6.7	100%	100%
42	Yoon^87^	2010	South Korea	Asian	NSCLC	1425	3011	56.28%	60.21%	57–63	56–62	51.23%	48.25%
					LUAD	1009	3011						
					LUSC	346	3011						
43	Zhao^88^	2013	China	Asian	LC	784	782	73.30%	71.60%	62.33 ± 10.74	62.72 ± 10.71	68.50%	52.30%
					LUAD	360	782						
					LUSC	253	782						
					LC Smoker	537	409						
					LC Non smoker	224	373						

*LC* Lung cancer, *NSCLC* non-small-cell lung carcinoma, *SCLC* small cell lung carcinoma, *LUAD* Lung adenocarcinoma, *LUSC* Lung squamous cell carcinoma.

Data are mean ± SD, or mean (IQR),or IQR, or n, unless otherwise stated.

**Table 3:** Basic features of the included study (2).

ID	Studies	Year	Genotyping methods	Type of LC	LC(n)					Controls(n)					LC vs.Controls OR [95% Cl]	Hardy–Weinberg
					AA	CA	CC	A	C	AA	CA	CC	A	C	C vs.A	PHWE
1	Bae^58^	2012	PCR	LC	402	501	191	1305	883	422	522	156	1366	834	1.11 [0.98, 1.25]	0.79
2	Brenner (Phase 1)^59^	2013	HumanHap	LC	–	–	–	–	–	–	–	–	–	–	1.22 [1.15, 1.29]	Yes
3	Brenner (Phase 2)^59^	2013	Illumina Chips	LC	–	–	–	–	–	–	–	–	–	–	1.10 [1.03, 1.16]	Yes
4	Broderick (Phase 1)^60^	2009	Illumina Chips	LC	–	–	–	–	–	–	–	–	–	–	0.97 [0.88, 1.07]	Yes
5	Broderick (Phase 2)^60^	2009	Illumina arrays	LC	–	–	–	–	–	–	–	–	–	–	0.95 [0.88, 1.03]	Yes
6	Chen^50^	2012	TaqMan	LC	45	101	50	191	201	69	112	48	250	208	1.26 [0.97, 1.66]	0.838
				LUAD	17	47	32	81	111	69	112	48	250	208	1.65 [1.17, 2.31]	
				LUSC	14	23	7	51	37	69	112	48	250	208	0.73 [0.47, 1.14]	
				SCLC	4	10	2	18	14	69	112	48	250	208	0.93 [0.45, 1.92]	
7	Cheng^61^	2016	Affymetrix Genome-Wide Array	LC	–	–	–	–	–	–	–	–	–	–	1.20 [1.11, 1.30]	0.3
8	Dong^14^	2017	Illumina Genome Analyzer	NSCLC	44	111	37	199	185	96	138	44	330	226	1.36 [1.04, 1.76]	0.631
9	Furuie^62^	2021	TaqMan and PCR	LC	172	216	74	560	364	137	171	71	445	313	0.92 [0.76, 1.12]	0.177
10	Hosgood^12^	2015	Illumina arrays	LC	447	909	374	1803	1657	508	646	195	1662	1036	1.47 [1.33, 1.63]	0.653
11	Hsiung^45^	2010	Illumina Chips	LC	599	1187	522	2385	2231	852	1132	337	2836	1806	1.47 [1.35, 1.60]	0.211
				LUAD	428	922	398	1778	1718	852	1132	337	2836	1806	1.52 [1.39, 1.66]	
				LUSC	60	82	35	202	152	852	1132	337	2836	1806	1.18 [0.95, 1.47]	
12	Hu^10^	2011	Affymetrix Genome-Wide Array	LC	2393	4294	1872	9080	8038	3231	4533	1614	10,995	7761	1.25 [1.20, 1.31]	0.724
				LUSC	896	1508	613	3300	2734	3231	4533	1614	10,995	7761	1.17 [1.11, 1.24]	
				LUAD	1148	2155	1020	4451	4195	3231	4533	1614	10,995	7761	1.34 [1.27, 1.41]	
				SCLC	231	405	144	867	693	3231	4533	1614	10,995	7761	1.13 [1.02, 1.26]	
				LC Smoker	1497	2490	1039	5484	4568	1327	1827	661	4481	3149	1.19 [1.12, 1.26]	0.455
				LC Non smoker	896	1804	833	3596	3470	1904	2706	953	6514	4612	1.36 [1.28, 1.45]	0.873
13	Ito^63^	2012	TaqMan and PCR	LC	248	340	128	836	596	279	329	108	887	545	1.16 [1.00, 1.35]	0.496
14	Jaworowska^64^	2011	TaqMan	LC	247	403	205	897	813	263	425	156	951	737	1.17 [1.02, 1.34]	0.494
15	Jin^65^	2009	PCR	NSCLC	353	627	232	1333	1091	450	658	231	1558	1120	1.14 [1.02, 1.27]	0.719
				LUAD	–	–	–	–	–	–	–	–	–	–	1.39 [1.13, 1.70]	
				LUSC	–	–	–	–	–	–	–	–	–	–	1.01 [0.78, 1.31]	
				NSCLC Smoker	–	–	–	–	–	–	–	–	–	–	1.11 [0.88, 1.40]	Yes
				NSCLC Non smoker	–	–	–	–	–	–	–	–	–	–	1.59 [1.21, 2.10]	Yes
16	Kohno^66^	2011	PCR	LUSC	142	175	53	459	281	116	165	39	397	243	1.00 [0.80, 1.24]	0.09
17	Lan^67^	2013	TaqMan	LC	43	109	41	195	191	70	103	24	243	151	1.58 [1.19, 2.10]	0.137
18	Lan^68^	2012	Illumina arrays	LC	–	–	–	5725	5285	–	–	–	5452	3634	1.38 [1.31, 1.47]	Yes
19	Landi^69^	2009	Illumina Chips	LC	–	–	–	5349	6129	–	–	–	5836	5860	1.09 [1.03, 1.15]	Yes
				LUAD	–	–	–	–	–	–	–	–	–	–	1.23 [1.13,1.33]	
				LUSC	–	–	–	–	–	–	–	–	–	–	1.01 [0.93, 1.10]	
				SCLC	–	–	–	–	–	–	–	–	–	–	1.00 [0.90, 1.13]	
				LC Smoker	–	–	–	–	–	–	–	–	–	–	1.06 [1.01, 1.12]	Yes
				LC Non smoker	–	–	–	–	–	–	–	–	–	–	1.34 [1.11, 1.61]	Yes
20	Li^70^	2012	Sequenom Mass Array iPLEX	LC	–	–	–	–	–	–	–	–	–	–	1.18 [1.09, 1.27]	0.49
21	Li^51^	2016	PCR	LC	109	201	81	419	363	117	159	61	393	281	1.21 [0.98, 1.49]	0.58
22	Liu^71^	2015	Sequenom Mass Array iPLEX	LC	72	139	77	283	293	92	173	52	357	277	1.33 [1.06, 1.67]	0.052
23	Machiela^72^	2015	Illumina arrays	LC	–	–	–	5675	5239	–	–	–	5419	3567	1.38 [1.30, 1.47]	Yes
24	Mandour^73^	2020	TaqMan	LC	6	12	22	24	56	3	19	18	25	55	1.06 [0.54, 2.08]	0.505
				NSCLC	5	11	20	21	51	3	19	18	25	55	1.10 [0.55, 2.21]	
				SCLC	0	0	2	0	4	3	19	18	25	55	4.14 [0.21,79.73]	
				LUAD	2	8	16	12	40	3	19	18	25	55	1.52 [0.68, 3.37]	
				LUSC	2	0	2	4	4	3	19	18	25	55	0.45 [0.11, 1.97]	
25	McKay^74^	2008	Illumina Chips	LC	–	–	–	–	–	–	–	–	–	–	1.18 [1.10, 1.26]	Yes
26	Miki^75^	2010	Illumina arrays	LUAD	622	1048	416	2292	1880	4093	5246	1695	13,432	8636	1.28 [1.19, 1.36]	0.835
27	Myneni^46^	2013	PCR	LC	122	141	89	385	319	157	212	78	526	368	1.18 [0.97, 1.45]	0.659
28	Pande^76^	2011	Illumina Chips	LC	–	–	–	1567	1795	–	–	–	1230	1240	1.14 [1.02, 1.26]	0.46
29	Seow^77^	2017	Illumina arrays, Affymetrix Genome-Wide Array, TaqMan and PCR	LUAD	–	–	–	7655	7355	–	–	–	7636	6504	1.13 [1.08, 1.18]	Yes
30	Shiraishi^78^	2016	TaqMan	LUAD	2057	3386	1387	7500	6160	5723	7133	2299	18,579	11,731	1.30 [1.25, 1.36]	0.323
31	Shiraishi^79^	2012	TaqMan	LUAD	1386	2265	997	5037	4259	4650	5856	1858	15,156	9572	1.34 [1.28, 1.40]	0.838
				LUAD smoker	662	1146	461	2470	2068	2244	2837	931	7325	4699	1.31 [1.22, 1.40]	0.488
				LUAD Non smoker	722	1114	532	2558	2178	1979	2429	774	6387	3977	1.37 [1.28, 1.47]	0.52
32	Truong^80^	2010	TaqMan	LC	538	836	312	1912	1460	775	1014	312	2564	1638	1.20 [1.09, 1.31]	0.506
				LC Smoker	–	–	–	–	–	–	–	–	–	–	1.20 [1.04, 1.38]	Yes
				LC Non smoker	–	–	–	–	–	–	–	–	–	–	1.27 [1.10, 1.46]	Yes
33	Truong^80^	2010	TaqMan	LC	1878	4526	2722	8282	9970	2853	5817	3142	11,523	12,101	1.15 [1.10, 1.19]	0.116
				LC Smoker	–	–	–	–	–	–	–	–	–	–	1.13 [1.08, 1.19]	Yes
				LC Non smoker	–	–	–	–	–	–	–	–	–	–	1.22 [1.09, 1.35]	Yes
34	Wang^81^	2014	PCR	NSCLC	455	764	333	1674	1430	549	780	276	1878	1332	1.20 [1.09, 1.33]	0.971
				LUAD	200	372	174	772	720	549	780	276	1878	1332	1.31 [1.16, 1.49]	
				LUSC	186	293	117	665	527	549	780	276	1878	1332	1.12 [0.98, 1.28]	
35	Wang^82^	2016	Mass Array	LC	131	257	112	519	481	178	242	80	598	402	1.38 [1.15, 1.65]	0.881
36	Wang^47^	2010	Illumina Chips	LC	42	115	82	199	279	136	259	158	531	575	1.29 [1.04, 1.61]	0.146
				SCLC	11	18	10	40	38	136	259	158	531	575	0.88 [0.55, 1.39]	
				NSCLC	31	97	72	159	241	136	259	158	531	575	1.40 [1.11, 1.77]	
				LUAD	13	60	39	86	138	136	259	158	531	575	1.48 [1.10, 1.99]	
				LUSC	8	23	17	39	57	136	259	158	531	575	1.35 [0.88, 2.06]	
37	Wei^39^	2015	TaqMan and PCR	NSCLC	190	353	159	733	671	814	1269	437	2897	2143	1.24 [1.10, 1.39]	0.13
38	Xing^83^	2016	TaqMan	NSCLC	216	164	38	596	240	268	124	18	660	160	1.66 [1.32, 2.09]	0.452
39	Yang^84^	2010	TaqMan	LC	–	–	–	–	–	–	–	–	–	–	1.11 [0.99, 1.24]	Yes
				LC Smoker	–	–	–	–	–	–	–	–	–	–	1.08 [0.92, 1.26]	Yes
				LC Non smoker	–	–	–	–	–	–	–	–	–	–	1.19 [0.98, 1.44]	Yes
40	Yin^85^	2014	TaqMan	LC	139	273	112	551	497	186	255	83	627	421	1.34 [1.13, 1.60]	0.777
				LUAD	84	196	85	364	366	186	255	83	627	421	1.50 [1.24, 1.81]	
41	Yoo^86^	2020	ARRAY iPLEX assay	LC	269	321	109	859	539	241	283	82	765	447	1.07 [0.92, 1.24]	0.94
42	Yoon^87^	2010	Affymetrix Genome-Wide Array	NSCLC	467	696	262	1630	1220	1186	1406	419	3778	2244	1.26 [1.15, 1.38]	0.944
				LUAD	313	497	199	1123	895	1186	1406	419	3778	2244	1.34 [1.21, 1.49]	
				LUSC	128	165	53	421	271	1186	1406	419	3778	2244	1.08 [0.92, 1.27]	
43	Zhao^88^	2013	TaqMan	LC	–	–	–	847	721	–	–	–	938	626	1.28 [1.11, 1.47]	0.61
				LUAD	–	–	–	–	–	–	–	–	–	–	1.98 [1.34, 2.93]	
				LUSC	–	–	–	–	–	–	–	–	–	–	1.32 [0.79, 2.19]	
				LC Smoker	–	–	–	–	–	–	–	–	–	–	1.52 [1.01, 2.28]	Yes
				LC Non smoker	–	–	–	–	–	–	–	–	–	–	1.79 [1.06, 3.03]	Yes

*LC* Lung cancer, *NSCLC* non-small-cell lung carcinoma, *SCLC* small cell lung carcinoma, *LUAD* Lung adenocarcinoma, *LUSC* Lung squamous cell carcinoma, *PCR* polymerase chain reaction, *PHWE P* value of Hardy-Wenberg equilibrium.

**Table 4:** Newcastle Ottawa scale (NOS).

Studies	Select				Comparability^a^	Expose			Total score^b^
	1	2	3	4	5	6	7	8	
	I	II	III	IV	V	VI	VII	VIII	
Bae 2012	☆	☆	☆	☆	☆☆	☆	☆		8☆
Brenner (Phase 1)2013	☆	☆	☆		☆☆	☆	☆		7☆
Brenner (Phase 2)2013	☆	☆	☆		☆☆	☆	☆		7☆
Broderick (Phase 1)2009	☆	☆	☆		☆☆	☆	☆		7☆
Broderick (Phase 2)2009	☆	☆	☆	☆	☆☆	☆	☆		8☆
Chen 2012	☆	☆	☆	☆	☆☆	☆	☆		8☆
Cheng 2016	☆	☆	☆	☆	☆☆	☆	☆		8☆
Dong 2017	☆	☆	☆	☆	☆☆	☆	☆		8☆
Furuie 2021	☆	☆	☆	☆	☆☆	☆	☆		8☆
Hosgood 2015	☆	☆	☆	☆	☆☆	☆	☆		8☆
Hsiung 2010	☆	☆	☆	☆	☆☆	☆	☆		8☆
Hu 2011	☆	☆	☆	☆	☆☆	☆	☆		8☆
Ito 2012	☆	☆	☆	☆	☆☆	☆	☆		8☆
Jaworowska 2011	☆	☆	☆	☆	☆☆	☆	☆		8☆
Jin 2009	☆	☆	☆	☆	☆☆	☆	☆		8☆
Kohno 2011	☆	☆	☆	☆	☆☆	☆	☆		8☆
Lan 2013	☆	☆	☆	☆	☆☆	☆	☆		8☆
Lan 2012	☆	☆	☆	☆	☆☆	☆	☆		8☆
Landi 2009	☆	☆	☆		☆☆	☆	☆		7☆
Li 2012	☆	☆	☆	☆	☆☆	☆	☆		8☆
Li 2016	☆	☆	☆	☆	☆☆		☆		7☆
Liu 2015	☆	☆	☆	☆	☆☆		☆		7☆
Machiela 2015	☆	☆	☆	☆	☆☆	☆	☆		8☆
Mandour 2020	☆	☆	☆	☆	☆☆	☆	☆		8☆
McKay 2008	☆	☆	☆		☆☆	☆	☆		7☆
Miki 2010	☆	☆	☆	☆	☆☆	☆	☆		8☆
Myneni 2013	☆	☆	☆	☆	☆☆	☆	☆		8☆
Pande 2011	☆	☆	☆	☆	☆☆	☆	☆		8☆
Seow 2017	☆	☆	☆	☆	☆☆	☆	☆		8☆
Shiraishi 2016	☆	☆	☆	☆	☆☆	☆	☆		8☆
Shiraishi 2012	☆	☆	☆	☆	☆☆	☆	☆		8☆
Truong (Asians) 2010	☆	☆	☆	☆	☆☆	☆	☆		8☆
Truong (Caucasians) 2010	☆	☆	☆	☆	☆☆	☆	☆		8☆
Wang 2014	☆	☆	☆	☆	☆☆	☆	☆		8☆
Wang 2016	☆	☆	☆	☆	☆☆	☆	☆		8☆
Wang 2010	☆	☆	☆	☆	☆☆	☆	☆		8☆
Wei 2015	☆	☆	☆	☆	☆☆	☆	☆		8☆
Xing 2016	☆	☆	☆	☆	☆☆	☆	☆		8☆
Yang 2010	☆	☆	☆	☆	☆☆	☆	☆		8☆
Yin 2014	☆	☆	☆	☆	☆☆	☆	☆		8☆
Yoo 2020	☆	☆	☆	☆	☆☆	☆	☆		8☆
Yoon 2010	☆	☆	☆	☆	☆☆	☆	☆		8☆
Zhao 2013	☆	☆	☆	☆	☆☆	☆	☆		8☆

^a^Two stars with the highest comparability; ^b^Full score is 9☆.1–8: Case–control studies (CC); I-VIII: Cohort studies (CS).

1: Case definition; 2: Demonstrations box; 3: Selection of control group; 4: Definition of control group; 5: Choose the most important/second most important factor; 6: Determination of exposure; 7: Methods for determining cases and control groups; 8: No response rate.

I: representativeness of exposure; II: selection of non-exposed persons; III: Determination of exposure; IV: proof of no interesting results at the beginning; V: comparability; VI: evaluation of results; VII: long enough follow-up time; VIII: adequacy of follow-up.

### Quantitative analysis

#### LC

**Figure 2:**
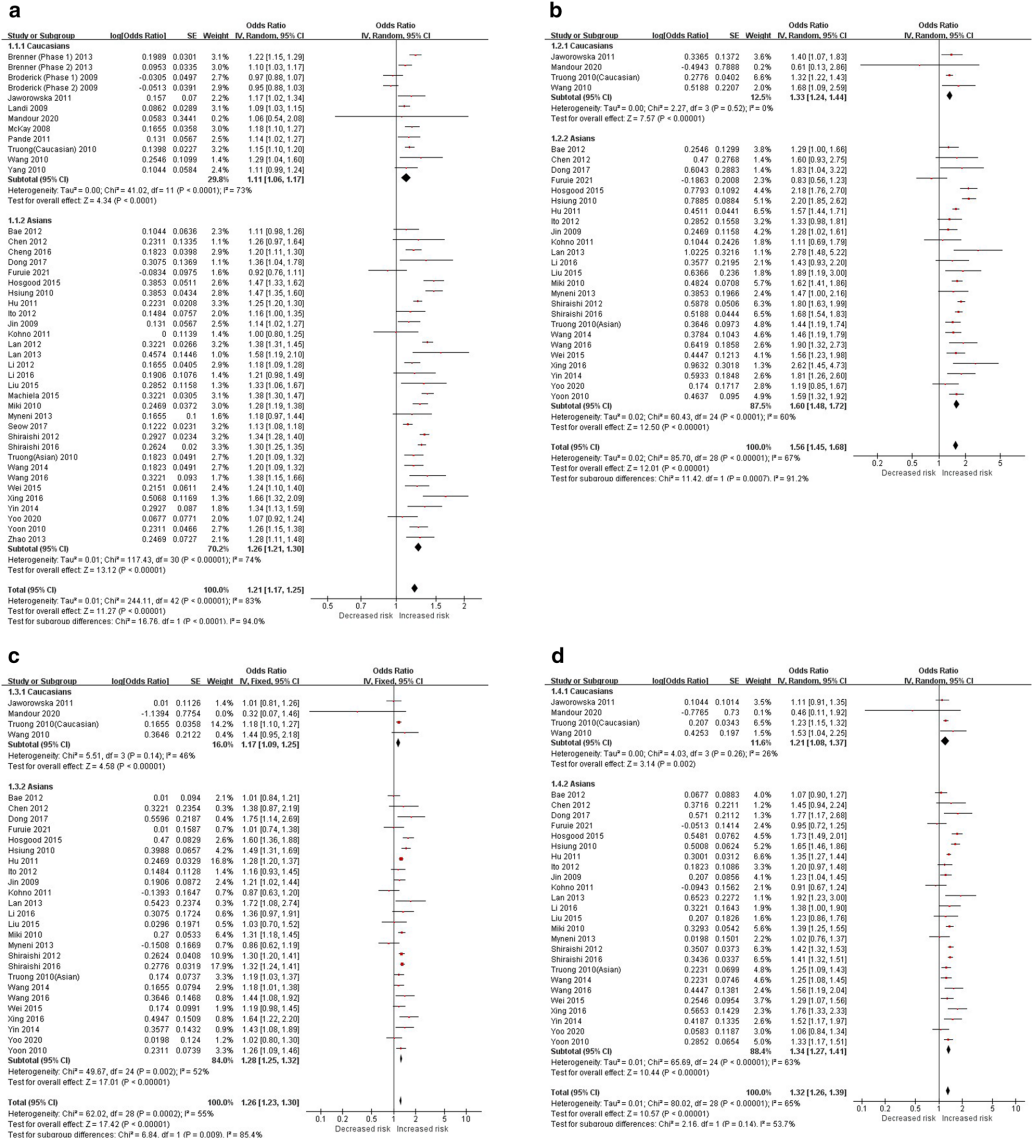

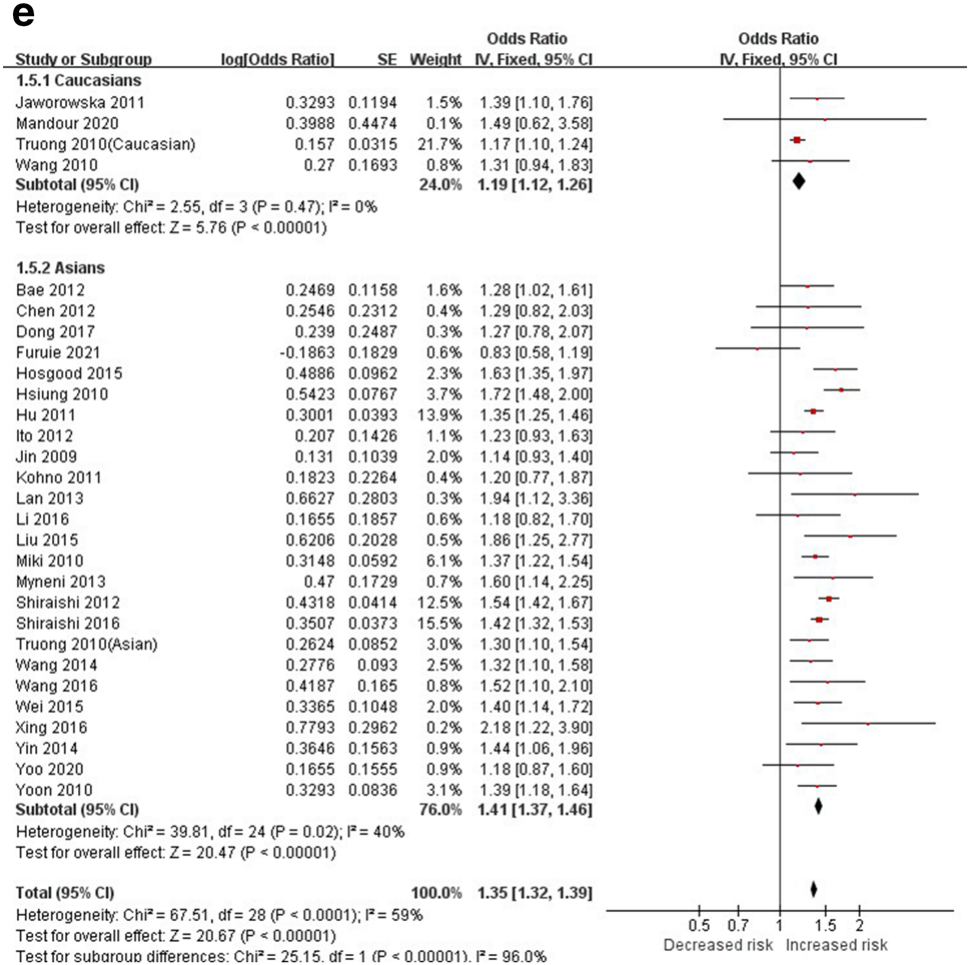
Forest plots of LC. (**a**) Forest plot of the allele genetic model (C vs. A) (Random). (**b**) Forest plot of the additive genetic model (CC vs. AA) (Random). (**c**) Forest plot of the heterozygous genetic model (CA vs. AA) (Fixed). (**d**) Forest plot of the dominant genetic model (CA + CC vs. AA) (Random). (e) Forest plot of the recessive genetic model (CC vs. AA + CA) (Fixed).

The allelic model (C vs. A) was used to evaluate the association of *TERT* 2736100 with LC susceptibility. The random effects model was used for analysis as the test results showed that there was heterogeneity after the heterogeneity test (Overall population: *P* < 0.00001, I^2^= 83%; Caucasians: *P* < 0.0001, I^2^ = 73%; Asians: *P* < 0.00001, I^2^ = 74%) (Fig. 2a, Table 5). It was found that the C allele was associated with the risk of LC (Overall population: [OR] = 1.21, 95%CI [1.17, 1.25]; Caucasians: [OR] = 1.11, 95%CI [1.06, 1.17]; Asians: [OR] = 1.26, 95%CI [1.21, 1.30]), and Asians had a higher risk of LC than Caucasians (C vs. A: Caucasians: [OR] = 1.11 /Asians: [OR] = 1.26) (Fig. 2a, Table 5). The additive, heterozygous, dominant and recessive genetic models (CC vs. AA, CA vs. AA, CA + CC vs. AA and CC vs. AA + CA) were further used to evaluate the correlation between *TERT* 2736100 and LC since 29 of the 43 studies reported complete genotype frequencies. And the fixed-effects model (*P* > 0.1 or I^2^ < 50%) and random-effects model (*P* < 0.1 or I^2^ > 50%) were used to analyze each subgroup due to the different heterogeneity of each subgroup. Meta-analysis showed that people with "C" genotype had higher risks of LC than those with "A" genotype (*P* < 0.00001), and Asians had higher risks of LC than Caucasians (CC vs. AA: Caucasians: [OR] = 1.33/Asians: [OR] = 1.60; CA vs. AA: Caucasians: [OR] = 1.17/Asians: [OR] = 1.26; CA + CC vs. AA: Caucasians: [OR] = 1.22/Asians: [OR] = 1.34; CC vs. AA + CA: Caucasians: [OR] = 1.19/Asians: [OR] = 1.41) (Fig. 2b–e, Table 5). It’s also found that carriers of the CC genotype ([OR] = 1.56) were more likely to develop LC than carriers of the CA genotype ([OR] = 1.25) (Table 5).

**Table 5:** The results of Meta-analysis and publication bias (LC).

Genetic model	Subgroup	Study (n)	Heterogeneity test		Sample		Model	OR [95% Cl]	Effect *P* value	Publication bias	
			*P* values	I^2^ (%)	Cases (n)	Controls (n)				*P*_Begg_	*P*_Egger_
Allele (C vs.A)	Caucasians	12	< 0.0001	73	73,886	81,138	Random	1.11 [1.06, 1.17]	< 0.0001	0.891	0.742
	Asians	31	< 0.00001	74	125,996	182,574	Random	1.26 [1.21, 1.30]	< 0.00001	0.865	0.55
	Total	43	< 0.00001	83	199,882	263,712	Random	1.21 [1.17, 1.25]	< 0.00001	0.843	0.489
Additive (CC vs.AA)	Caucasians	4	0.52	0	5204	6729	Fixed	1.33 [1.24, 1.44]	< 0.00001	1.000	0.919
	Asians	25	< 0.0001	60	19,719	35,876	Random	1.60 [1.48, 1.72]	< 0.00001	1.000	0.436
	Total	29	< 0.00001	67	24,923	42,605	Random	1.56 [1.45, 1.68]	< 0.00001	1.000	0.575
Heterozygous (CA vs.AA)	Caucasians	4	0.14	46	7229	9775	Fixed	1.17 [1.09, 1.25]	< 0.00001	0.497	0.496
	Asians	25	0.002	52	31,075	57,920	Random	1.26 [1.20, 1.33]	< 0.00001	0.513	0.353
	Total	29	0.0002	55	38,304	67,695	Random	1.25 [1.19, 1.31]	< 0.00001	0.485	0.223
Dominant (CA + CC vs.AA)	Caucasians	4	0.26	26	10,260	13,249	Fixed	1.22 [1.15, 1.30]	< 0.00001	0.497	0.650
	Asians	25	< 0.00001	63	39,133	68,537	Random	1.34 [1.27, 1.41]	< 0.00001	0.815	0.356
	Total	29	< 0.00001	65	49,393	81,786	Random	1.32 [1.26, 1.39]	< 0.00001	0.78	0.281
Recessive (CC vs.AA + CA)	Caucasians	4	0.47	0	10,260	13,249	Fixed	1.19 [1.12, 1.26]	< 0.00001	1.000	0.138
	Asians	25	0.02	40	39,133	68,537	Fixed	1.41 [1.37, 1.46]	< 0.00001	0.64	0.524
	Total	29	< 0.0001	59	49,393	81,786	Random	1.37 [1.30, 1.45]	< 0.00001	0.641	0.172

#### LC subtypes

A further stratified analysis of these LC studies was performed since there were four different disease types in LC studies: Non-small-cell lung carcinoma(NSCLC, N = 21), Small cell lung carcinoma (SCLC, N = 7), Lung adenocarcinoma(LUAD, N = 17) and Lung squamous cell carcinoma(LUSC, N = 13). Meta-analysis of the allele model (C vs. A) found that the C allele was associated with the risk of NSCLC (Overall population: [OR] = 1.27, 95%CI [1.22, 1.33]; Caucasians: [OR] = 1.19, 95%CI [1.09, 1.31]; Asians: [OR] = 1.28, 95%CI [1.22, 1.34]), and Asians had a higher risk of NSCLC than Caucasians (C vs. A: Caucasians: [OR] = 1.19/Asians: [OR] = 1.28) (Fig. S1 in supplemental content, Table 6). In SCLC patients, the C allele was associated with the risk of SCLC only in Asians (Overall population: [OR] = 1.03, 95%CI [0.98, 1.09]; Caucasians: [OR] = 1.00, 95%CI [0.94, 1.06]; Asians: [OR] = 1.11, 95%CI [1.01, 1.22]) (Fig. S1 in supplemental content, Table 6). In LUAD patients, the C allele was associated with the risk of developing LUAD (Overall population: [OR] = 1.32, 95%CI [1.26, 1.38]; Caucasians: [OR] = 1.22, 95%CI [1.16, 1.28]; Asians: [OR] = 1.34, 95%CI [1.27, 1.41]), and Asians had a higher risk of developing LUAD than Caucasians (C vs. A: Caucasians: [OR] = 1.22/Asians: [OR] = 1.34) (Fig. S2 in supplemental content, Table 6). In LUSC patients, the C allele was associated with LUSC risk in Asians but not in Caucasians (Overall population: [OR] = 1.09, 95%CI [1.06, 1.13]; Caucasians: [OR] = 1.04, 95%CI [0.99, 1.10]; Asians: [OR] = 1.13, 95%CI [1.08, 1.18]) (Fig. S2 in supplemental content, Table 6). It’s also found that NSCLC patients ( [OR] = 1.27) had a stronger disease association than SCLC patients ( [OR] = 1.03) when the OR values were compared (Fig. S1 in supplemental content, Table 6), and LUAD patients ([OR] = 1.32) had a stronger disease association than LUSC patients ([OR] = 1.09) (Fig. S2 in supplemental content, Table 6).

**Table 6:** The results of Meta-analysis and publication bias (Allele genetic model, C vs.A).

Type	Subgroup	Study (n)	Heterogeneity test		Sample		Model	OR [95% Cl]	Effect *P* value	Publication bias	
			*P* values	I^2^ (%)	Cases (n)	Controls (n)				*P*_Begg_	*P*_Egger_
LC (NSCLC and SCLC)											
NSCLC	Total	21	< 0.00001	72	96,290	177,388	Random	**1.27 [1.22, 1.33]**	** < 0.00001**	**1.000**	**0.778**
	Caucasians	4	0.48	0	18,968	36,506	Fixed	**1.19 [1.09, 1.31]**	**0.0001**	**0.497**	**0.862**
	Asians	17	< 0.00001	76	77,322	140,882	Random	**1.28 [1.22, 1.34]**	** < 0.00001**	**0.869**	**0.59**
SCLC	Total Caucasians	7	0.51	0	5658	49,424	Fixed	1.03 [0.98, 1.09]	0.24	**0.293**	**0.939**
		4	0.76	0	3848	26,008	Fixed	1.00 [0.94, 1.06]	0.96	**1.000**	**0.644**
	Asians	3	0.65	0	1810	23,416	Fixed	**1.11 [1.01, 1.22]**	**0.03**	**0.602**	**0.243**
	Total (NSCLC and SCLC)	28	< 0.00001	79	101,948	226,812	Random	**1.22 [1.17, 1.28]**	** < 0.00001**	**0.836**	**0.804**
NSCLC (LUAD and LUSC)											
LUAD	Total	17	< 0.00001	77	73,546	170,050	Random	**1.32 [1.26, 1.38]**	** < 0.00001**	**0.249**	**0.083**
	Caucasians	4	0.53	0	10,838	36,214	Fixed	**1.22 [1.16, 1.28]**	** < 0.00001**	**0.174**	**0.113**
	Asians	13	< 0.00001	80	62,708	133,836	Random	**1.34 [1.27, 1.41]**	** < 0.00001**	**0.222**	**0.089**
LUSC	Total	13	0.04	45	18,216	78,688	Fixed	**1.09 [1.06, 1.13]**	** < 0.00001**	**1.000**	**0.218**
	Caucasians	4	0.31	16	7228	36,506	Fixed	1.04 [0.99, 1.10]	0.12	**0.497**	**0.897**
	Asians	9	0.12	38	10,988	42,182	Fixed	**1.13 [1.08, 1.18]**	** < 0.00001**	**0.404**	**0.061**
	Total **(**LUAD and LUSC**)**	30	< 0.00001	82	91,762	248,738	Random	**1.23 [1.17, 1.29]**	** < 0.00001**	**0.339**	**0.982**

Significance values are in Bold.

#### Analysis of smoking status in LC patients

Among the included studies, 25 reported smoking or non-smoking in LC patients, of which 9 reported smoking history in LC patients and 16 reported no smoking history in LC patients. Therefore, a stratified analysis of smoking in LC patients in these 25 studies was conducted to clarify whether smoking caused variation in *TERT* rs2736100 and increased the risk of LC. Meta-analysis of the allele model (C vs. A) found that the C allele was associated with the risk of LC in both the smoking group and the non-smoking group (Smoking: [OR] = 1.16, 95%CI [1.09, 1.23]; Non-smoking: [OR] = 1.34, 95%CI [1.26, 1.41]), and the risk of LC in the non-smoking group was higher than that in the smoking group (C vs. A: Smoking: [OR] = 1.16/Non-smoking: [OR] = 1.34), and it was also found that non-smokers had the highest risk of LC in Asians ([OR] = 1.36, 95%CI [1.27, 1.46]) (Fig. S3 in supplemental content, Table 7).

**Table 7:** Meta-analysis results of smoking status (Allele genetic model, C vs. A).

Type	Subgroup	Smoking status	Study (n)	Heterogeneity test		Sample		Model	OR [95% Cl]	Effect *P* value
				*P* values	I^2^ (%)	Cases (n)	Controls (n)			
LC	Overall	Smoking	9	< 0.00001	72	50,138	47,782	Random	**1.16 [1.09, 1.23]**	** < 0.00001**
		Non-smoking	16	< 0.00001	88	64,328	80,038	Random	**1.34 [1.26, 1.41]**	** < 0.00001**
		Total	25	< 0.00001	85	114,466	127,820	Random	**1.27 [1.20, 1.33]**	** < 0.00001**
	Caucasians	Smoking	3	0.16	45	29,540	23,384	Fixed	**1.10 [1.06, 1.13]**	** < 0.00001**
		Non-smoking	5	0.87	0	3808	13,182	Fixed	**1.24 [1.15, 1.35]**	** < 0.00001**
		Total	8	0.07	46	33,348	36,566	Fixed	**1.12 [1.08, 1.15]**	** < 0.00001**
	Asians	Smoking	6	0.10	46	20,598	24,398	Fixed	**1.22 [1.17, 1.27]**	** < 0.00001**
		Non-smoking	11	< 0.00001	85	60,520	66,856	Random	**1.36 [1.27, 1.46]**	** < 0.00001**
		Total	17	< 0.00001	81	81,118	91,254	Random	**1.31 [1.24, 1.38]**	** < 0.00001**
NSCLC	Overall	Smoking	3	0.05	67	11,894	22,070	Random	**1.20 [1.05, 1.36]**	**0.007**
		Non-smoking	8	< 0.0001	79	26,120	35,676	Random	**1.33 [1.18, 1.50]**	** < 0.00001**
		Total	11	< 0.0001	75	38,014	57,746	Random	**1.28 [1.18, 1.39]**	** < 0.00001**
	Caucasians	Smoking	1	–	–	5784	8850	Fixed	1.11 [0.98, 1.26]	0.10
		Non-smoking	3	0.60	0	924	3990	Fixed	**1.32 [1.08, 1.63]**	**0.007**
		Total	4	0.38	3	6708	12,840	Fixed	**1.16 [1.05, 1.30]**	**0.005**
	Asians	Smoking	2	0.18	44	6110	13,220	Fixed	**1.29 [1.21, 1.38]**	** < 0.00001**
		Non-smoking	5	< 0.00001	87	25,196	31,686	Random	**1.35 [1.17, 1.55]**	** < 0.0001**
		Total	7	< 0.00001	83	31,306	44,906	Random	**1.31 [1.18, 1.44]**	** < 0.00001**
SCLC	Caucasians	Smoking	1	–	–	1336	8850	Fixed	0.99 [0.88, 1.11]	0.87
		Non-smoking	3	0.25	27	100	3990	Fixed	1.04 [0.68, 1.59]	0.86
		Total	4	0.42	0	1436	12,840	Fixed	0.99 [0.89, 1.11]	0.91
LUAD	Overall	Smoking	2	0.13	57	7572	20,874	Random	**1.26 [1.16, 1.37]**	** < 0.00001**
		Non-smoking	7	< 0.00001	88	24,654	34,184	Random	**1.37 [1.20, 1.56]**	** < 0.00001**
		Total	9	< 0.00001	85	32,226	55,058	Random	**1.33 [1.22, 1.46]**	** < 0.00001**
	Caucasians	Smoking	1	–	–	3034	8850	Fixed	**1.20 [1.10, 1.31]**	** < 0.0001**
		Non-smoking	3	0.87	0	682	3990	Fixed	**1.40 [1.17, 1.68]**	**0.0002**
		Total	4	0.45	0	3716	12,840	Fixed	**1.24 [1.14, 1.34]**	** < 0.00001**
	Asians	Smoking	1	–	–	4538	12,024	Fixed	**1.31 [1.22, 1.41]**	** < 0.00001**
		Non-smoking	4	< 0.00001	94	23,972	30,194	Random	**1.36 [1.16, 1.59]**	**0.0001**
		Total	5	< 0.00001	92	28,510	42,218	Random	**1.35 [1.19, 1.52]**	** < 0.00001**
LUSC	Overall	Smoking	1	–	–	2750	8850	Fixed	1.03 [0.87, 1.22]	0.73
		Non-smoking	4	0.23	30	504	8632	Fixed	1.14 [0.95, 1.37]	0.16
		Total	5	0.30	19	3254	17,482	Fixed	1.08 [0.95, 1.22]	0.22
	Caucasians	Smoking	1	–	–	2750	8850	Fixed	1.03 [0.87, 1.22]	0.73
		Non-smoking	3	0.14	49	150	3990	Fixed	1.05 [0.75, 1.48]	0.77
		Total	4	0.26	24	2900	12,840	Fixed	1.03 [0.89, 1.20]	0.66
	Asians	Non-smoking	1	–	–	354	4642	Fixed	1.18 [0.95, 1.47]	0.13

Significance values are in Bold.

A further stratified analysis of the smoking status of patients with different types of LC was performed due to the presence of different types of LC in the included studies. For NSCLC, the *TERT* polymorphism (C vs. A) was associated with the risk of NSCLC in both smoking group and non-smoking group (Smoking: [OR] = 1.20, 95%CI [1.05, 1.36]; Non-smoking: [OR] = 1.33, 95%CI [1.18, 1.50]), and the non-smoking group had a higher risk of NSCLC than the smoking group (C vs. A: Smoking: [OR] = 1.20/Non-smoking: [OR] = 1.33), and it’s also found that non-smokers had the highest risk of NSCLC in Asians ( [OR] = 1.35, 95%CI [1.17, 1.55]) (Fig. S4 in supplemental content, Table 7). For LUAD, the *TERT* polymorphism (C vs. A) was associated with the risk of LUAD in both the smoking group and the non-smoking group (Smoking: [OR] = 1.26, 95%CI [1.16, 1.37]; Non-smoking: [OR] = 1.37, 95%CI [1.20, 1.56]), and the risk of developing LUAD in the non-smoking group was higher than that in the smoking group (C vs. A: Smoking: [OR] = 1.26/Non-smoking: [OR] = 1.37). In addition, the risk of LUAD was found to be the highest among non-smokers in Caucasians ( [OR] = 1.40, 95%CI [1.17, 1.68]) (Fig. S5 in supplemental content, Table 7). For LUSC and SCLC, *TERT* polymorphisms (C vs. A) were not associated with the risk of LUSC and SCLC in both smoking group and non-smoking group in all populations (*P* > 0.05) (Table 7).

### Sensitivity analysis

For LC, the sensitivity analysis results of the allele, additive, heterozygous, dominant and recessive genetic models (C vs. A, CC vs. AA, CA vs. AA, CA + CC vs. AA and CC vs. AA + CA) showed that none of the studies had significant sensitivity, indicating that there’s no significant difference in the result of the meta-analysis after removing any study (Fig. S6, Tables S2–S6 in supplemental content). For NSCLC, SCLC, LUAD and LUSC, the sensitivity analysis of the allele model (C vs. A) also showed no significant sensitivity (Fig. S7, Tables S7, S8 in supplemental content).

### Heterogeneity analysis

For LC, there was some heterogeneity in the overall population analysis results for the allele, additive, heterozygous, dominant and recessive genetic models (C vs. A, CC vs. AA, CA vs. AA, CA + CC vs. AA and CC vs. AA + CA) (*P* < 0.1 or I^2^> 50%), and this heterogeneity mainly exists in Asians (Table 5). In the stratified analysis, the allele model (C vs. A) of NSCLC and LUAD analysis results in the overall population also showed a certain degree of heterogeneity (*P* < 0.1 or I^2^> 50%), and this heterogeneity mainly existed in Asians (Table 6).

### Publication bias

For LC, the funnel plots of the allele, additive, heterozygous, dominant and recessive genetic models (C vs. A, CC vs. AA, CA vs. AA, CA + CC vs. AA and CC vs. AA + CA) were all roughly symmetrical, suggesting there’s no apparent bias (Fig. S8 in supplemental content). In terms of NSCLC, SCLC, LUAD and LUSC, the funnel plots of the allele model (C vs. A) were all roughly symmetrical (Fig. S9 in supplemental content). Additionally, the results of publication bias for all genetic models suggested that there were no obvious biases (*P*_Begg_ > 0.05, *P*_Egger_ > 0.05) (Tables 5, 6/Figs. S10–S12 in supplemental content).

### Trial sequential analysis (TSA)

For LC, TSA analysis of the allele, additive, heterozygous, dominant and recessive genetic models (C vs. A, CC vs. AA, CA vs. AA, CA + CC vs. AA and CC vs. AA + CA) showed Z-curve (blue line) crossed both the traditional boundary (green dashed line) and the TSA boundary (red line) (Figs. S13–S17 in supplemental content). In terms of NSCLC, SCLC, LUAD and LUSC, TSA analysis of the allele model (C vs. A) in the overall and Asian populations also showed the same results (Figs. S18–S21 in supplemental content). Similar results were found in TSA analysis of the allele model (C vs. A) for patients with LC, NSCLC, and LUAD in terms of smoking status (Figs. S22–S24 in supplemental content). These results showed the overall stability and credibility of the results of this meta-analysis. The TSA results of NSCLC, SCLC, LUAD and LUSC in Caucasians cannot be comprehensively analyzed due to the reasons such as small sample size or the absence of complete gene frequencies in some of the original data reported in the literature. In addition, TSA results for smoking status in SCLC and LUSC couldn’t be comprehensively analyzed because of these reasons as well.

### Summary of all the results

**Figure 3:**
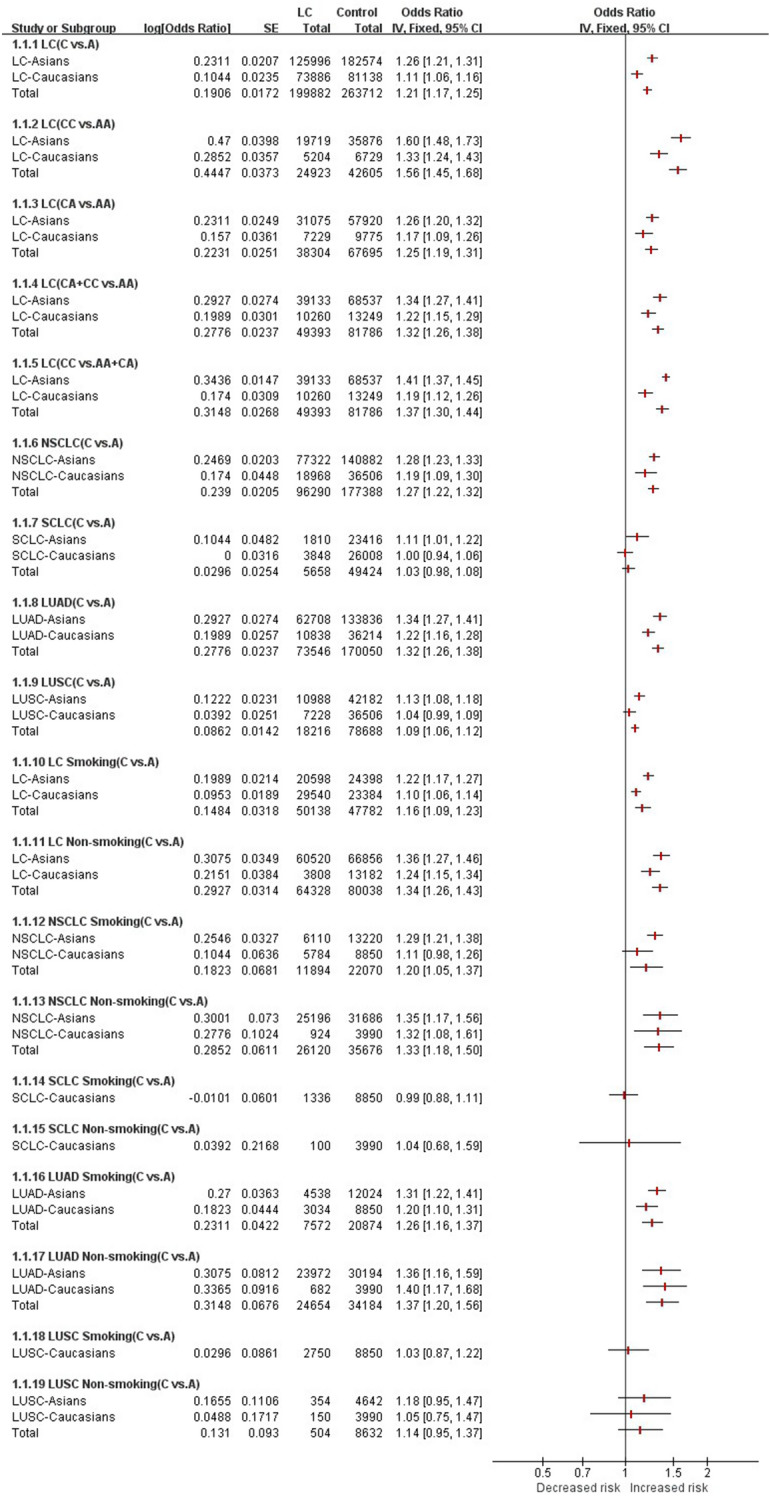
Summary forest plot of all results.

Due to the large amount of data in this study, a summative forest plot of all the results was created to show the statistical results more visually and more clearly, see Fig. 3.

## Discussion

Current studies have reported that gene polymorphisms in *TERT* and *TERC* are associated with telomere length^33–35^, and longer telomeres length contributes to an increased risk of LC^36–38^. The increased telomere length of the C allele of the rs2736100 (A > C) polymorphism in the second intron of *TERT* is related to cancer^44^. A number of research reports have also reported that the frequency of the C allele of *TERT* rs2736100 increases in patients with LC^9,45–48^. It’s showed that the C allele can upregulate the expression of *TERT*, maintain and prolong telomere length, thereby increasing the risk of LC. However, due to the existence of factors such as ethnic differences, different types of LC, environmental pollution and smoking, the association between *TERT* rs2736100 polymorphism and LC still lacks a unified conclusion. This study included the data of GWAS and case–control studies on the association of rs2736100 polymorphism with LC that have been reported so far to clarify the association between this polymorphism and LC and the differences in the association between different ethnic groups and different types of LC.

43 studies (including 99,941 LC patients and 131,856 healthy controls) were included in this meta-analysis. The association of *TERT* polymorphisms with LC susceptibility was first evaluated by using the allele, additive, heterozygous, dominant and recessive genetic models (C vs. A, CC vs. AA, CA vs. AA, CA + CC vs. AA and CC vs. AA + CA). And the results showed that the C allele and "C" genotype were associated with the risk of LC comparing with the A allele and "A" genotype in the overall population. These results are consistent with those of previous GWAS studies^10,12,14,45,47,50,59,61,64,65,68,69,74,76,77,79,80,87,88^. It indicates that people with C allele are more likely to suffer from LC, and C allele and "C" genotype are the risk factors for LC, and the C allele increases the risk of LC by extending telomere length. However, there are some GWAS that haven’t found the association between the C allele and LC^58–60,63^. The reasons for these different results may also be related to different ethnicities, countries, research methods, sample sizes, LC types, and linkage disequilibrium patterns. Previous studies also reported that the impact of *TERT* variation in Asians was stronger than that in Caucasians^45,55^. Another study showed that rs2735947 was the most significant SNP in the Caucasians rather than rs2736100^49^. Our findings also confirmed that the C allele and "C" genotype frequencies were indeed higher in Asians than in Caucasians, suggesting that Asians may have longer telomeres that leads to an increased risk of LC.

Since telomere length can vary with the histological type of LC^40,41^, different types of LC may have different degrees of association with *TERT* gene polymorphism due to their different pathological types. Therefore, a stratified analysis of the included LC studies was performed. Previous studies have found that longer telomere length contributes to increase the risk of LC, especially for NSCLC and LUAD^36–38^, and the C allele can increase the risk of NSCLC^65,83^. The results of our study also suggested that the C allele was associated with the risk of NSCLC. It indicates that the population carrying the C allele are more susceptible to NSCLC due to telomere lengthening. And it’s also found in our study that Asians had a higher risk of NSCLC than Caucasians, proving that Asians may have longer telomeres, which contribute to an increased risk of NSCLC. Some studies^47,69^ found that *TERT* rs2736100 wasn’t associated with the risk of SCLC in Caucasians, but Hu et al^10^ found that *TERT* rs2736100 could increase the risk of developing SCLC in Asians. Our results showed that the C allele was only associated with the risk of SCLC in Asians. It suggests that the C allele is a risk factor for SCLC in Asians but not in Caucasians, and the reason may be strongly related to the fact that Asian populations may have longer telomeres. When the OR values of NSCLC and SCLC were compared, it was found that NSCLC patients had a stronger disease association than SCLC patients. A previous study^92^ identified a locus on chromosome 5p15.33 that was significantly associated with the risk of LUAD in NSCLC, but not with other major histological types. Another study found that *TERT* s2736098 was significantly associated with an increased risk of SCLC in the Chinese population instead of rs2736100^89^. These findings, combined with our results, suggested that Asian populations and NSCLC patients may have longer telomeres, which triggered the risk of cancer, and *TERT* rs2736100 is of a higher value as a genetic marker for diagnosing the pathogenesis of NSCLC than SCLC.

NSCLC is the most common type of LC, and LUAD is the most prevalent subtype of NSCLC^73^. Previous studies^90^ have found rs2736100 to be a risk factor associating with increased susceptibility to LC, especially for LUAD. results of this study also showed that the C allele was associated with the risk of LUAD, confirming that the risk of developing LUAD is also strongly associated with telomere lengthening^36–38^. The results of this study also showed that Asians had a higher risk of LUAD than Caucasians, suggesting that Asians may possess longer telomeres, which contribute to an increased risk of LUAD. Some studies have found that there’s no such a risk association among LUSC patients^49^. Several other studies^47,69^ also showed that *TERT* rs2736100 wasn’t associated with the risk of developing LUSC in Caucasians. However, in some studies on Asians^10^, the C allele of *TERT* rs2736100 was found to increase the risk of developing LUSC. Results of this study also showed that the C allele was associated with LUSC risk in Asians but not Caucasians. It proves that the C allele is a risk factor for LUSC in Asians but not in Caucasians and the reason has a lot to do with the fact that Asian populations may has longer telomeres. It’s found that patients with LUAD had a stronger disease association than patients with LUSC. Previous studies have confirmed that rs2736100 was more associated with LUAD than with LUSC^69,91^, which is consistent with our findings. Similarly, there are studies^92^ have identified a locus on chromosome 5p15.33 that is clearly associated with the risk of LUAD but not with other major histological types. These evidences demonstrate that Asian populations and patients with LUAD may have longer telomeres, thereby triggering the risk of cancer, and *TERT* rs2736100 has a higher value as a genetic marker for diagnosing the pathogenesis of LUAD than LUSC.

Epidemiological surveys showed that although smoking was identified as a major environmental risk factor for LC worldwide, only a small proportion of smokers develop LC during their lifetime. In contrast, a large proportion of LC cases have no history of smoking^93,94^. LC in never-smokers differs from LC in smokers, and a large proportion of LC patients in never-smokers carry genetic variants in oncogenes^95^. Recent studies have shown that the genetic susceptibility of never-smokers to LC is associated with genetic variants with pan-cancer risk effects, and that gene-environment interactions are important in LC etiology^96^. Tumor suppressor genes are normally expressed in healthy cells due to key regulators of cell division, such as cyclin and cyclin-dependent kinases, as well as other cell cycle checkpoints that limit this process^97^. However, when oncogenes triggered by environmental factors are activated and tumor suppressor genes are turned off, the control of cell division is altered, and cancer starts from a single cell^98,99^. Studies have shown that multiple environmental risk factors such as smoking, heavy alcohol consumption, high intake of red meat and fat, low fiber intake , indoor and outdoor air pollution, and exposure to chemicals and radiation can contribute to genomic instability^100–104^. Genomic instability leads to nucleotide dysfunction, such as base substitution, base loss, nucleotide deletion, insertion or amplification of base pairs, which further induce DNA breaks, chromosomal remodeling or translocation. And if the damage is not fixed, it can lead to irreversible cell mutation and continuous growth^105,106^. In LC studies, CT and TT genotype carriers of miR-26a-1 rs7372209 and miR-16-1 rs1022960 who have been exposed to cooking fumes have a higher risk of LC than those who have not been exposed^107^. Another study evaluating the association between gene-radon interactions among uranium miners and LC indicated that the OR interaction effect of SNP rs6891344 and rs11747272 with chromosomes 5q23.2 was estimated to be 3.9 and 3.4, suggesting that uranium miners exposed to the radioactive gas radon are more susceptible to LC^108^. These evidences suggest that a variety of environmental factors other than smoking can also cause genetic variants that lead to LC. Therefore, a stratified analysis on the smoking status of LC patients included in the study was conducted to clarify whether smoking or non-smoking caused variation in *TERT* rs2736100 and increased the risk of LC. The results showed that the C allele was associated with the risk of LC in both smokers and non-smokers, and the risk of LC in non-smokers was higher than that in smokers. It’s been reported that rs2736100 is the most significant variation among non-smokers, while rs2736100 is less significant than rs36019446^49^ among smokers, which confirms that *TERT* variation has a stronger impact on non-smokers than on smokers^45,109^.A study also showed that *TERT* SNP was a risk factor for LC in never smokers^110^. Similarly, a case–control study also showed that the C allele increased the risk of LC in never smokers^111^. Therefore, smoking is not the most critical factor to cause variation in *TERT* rs2736100 and increase the risk of LC.

To further clarify this genetic difference between smokers and non-smokers, we performed a stratified analysis of different types of LC in different ethnic groups as the telomere length and the frequency of *TERT* gene variants were different in different ethnic groups and different histological types of LC^40,41^. The results of this study also showed that *TERT* polymorphism (C vs. A) was associated with the risk of NSCLC in both smokers and non-smokers, and the risk of NSCLC in non-smokers was higher than that in smokers. For LUAD, the same result existed: *TERT* polymorphism (C vs. A) was associated with the risk of LUAD in both smokers and non-smokers, and the risk of LUAD in non-smokers was higher than that in smokers. Previous studies have also found that there are non-tobacco related risk factors in the pathogenesis of NSCLC. These possible risk factors include: the exposure to cooking fume, hormones and viral infection^112^. Subramanian^113^ mentioned before that LUAD was the most common type among never smokers. Therefore, non-smokers are more likely to be at the risk of NSCLC and LUAD due to variation in *TERT* rs2736100 leading to telomere lengthening. It’s confirmed that smoking does cause variation in *TERT* rs2736100, which increases the risk of most LC (NSCLC, LUAD), however, it’s not the most critical factor. Evidence shows that^82^ education level, BMI, prior diagnosis of COPD, occupational exposure to pesticides, duration of smoking, exposure to a large number of cooking emissions, dietary factors (including less fish and shrimp, vegetables, soy products and nuts) and the excessive intake of meat in LC patients are all related to the development of LC. When combined with many environmental and lifestyle factors, *TERT* rs2736100 is still significantly associated with LC^82^. Therefore, LC (NSCLC, LUAD) is a multi-etiological disease caused by a combination of genetic and lifestyle factors. Comparing with different ethnic groups, it’s found that the risk of LC and NSCLC in the non-smokers was the highest in Asians. Combined with the results above, it’s proved that the Asian non-smoking populations may be more likely at the risk of LC and NSCLC due to the elevated frequency of *TERT* rs2736100 C allele combined with environmental factors that cause telomere lengthening. But for LUAD, non-smokers were found to have the highest risk of developing LUAD in Caucasians rather than Asians. The reason for this is still related to the small sample size of non-smokers in Caucasians, and the fact that there’s not only one pathological type of LUAD in NSCLC but also many other types such as LUSC and large cell lung cancer (LCLC), which can lead to inconsistent results in the analysis of NSCLC and LUAD. In addition, the majority of non-smoking LUAD patients included in this study are Asian females (Asian females: N = 9618/Overall: N = 12,327), indicating that non-smoking females in the Asians are more likely to have the risk of LUAD. Previous studies have also confirmed that LUAD is more common in females^114,115^. Patel et al. showed that among the never-smoking LC patients, the number of females exceeded that of males^116^. There was evidence confirmed that the common genetic variation of *TERT-CLPTM1L* was associated with the risk of LUAD in non-smoking Asian females^45^. This can be explained by the following assumptions: females are more likely to be exposed to second-hand smoking, and exposed to coal for cooking at home and hormone replacement therapy. All these reasons can lengthen telomere to avoid apoptosis and ultimately lead to cancer^117^.

For LUSC and SCLC, *TERT* polymorphisms (C vs. A) were not associated with the risk of them in all populations, both in smokers and in non-smokers. Therefore, smoking may not cause variation in *TERT* rs2736100 that increase the risk of LUSC and SCLC. The cause of variation in *TERT* rs2736100 leading to LUSC and SCLC remains to be further clarified.

Limitations of this study: ① This meta-analysis is based on the research reports of different ethnic groups and different types of LC, which will inevitably produce some heterogeneity; ② The methods of gene detection and genotyping used in all studies were different, and there will be some differences in data results; ③ In terms of sample size, this study is sufficient in general. However, after subgroup analysis according to different LC types and ethnicity, the results signify that the sample size of SCLC and LUSC is still small. This will inevitably produce some false negative results for SCLC and LUSC; ④ Although this study discussed the effects of smoking, environment, lifestyle and other factors on LC in details, from the perspective of smoking status, the sample size of smoking patients reported in these studies is still relatively small, especially those of SCLC and LUSC studies. Therefore, to some extent, the reliability of the results of the correlation between smoking and the risk of SCLC and LUSC will be affected; ⑤ All the literatures included in this study are in English, not in the other languages.

## Conclusion

In conclusion, the C allele of *TERT* rs2736100 is a risk factor for LC, NSCLC, and LUAD in different ethnic groups, and the risk is more common in Asians. Moreover, the C allele is a risk factor for LUSC and SCLC in Asians but not in Caucasians. Among the different types of LC, NSCLC patients have stronger risk correlation than SCLC patients, and LUAD patients have a stronger disease risk correlation than LUSC patients. Asians have a more common risk of various types of LC because they may have longer telomeres than Caucasians. The C allele is correlated with the risk of LC, NSCLC and LUAD in smokers and non-smokers, and the risk of LC in non-smokers of different ethnic groups is more common than that in smokers. In the Asians, non-smoking females are more at the risk of developing LUAD. Therefore, smoking does cause variation in *TERT* rs2736100 and increases the risk of most LC (NSCLC, LUAD), but it’s not the most critical factor.

LC (NSCLC, LUAD) is a multi-etiological disease caused by a combination of genetic, environmental and lifestyle factors. Of course, it’s necessary to integrate and analyze the data of studies with a larger sample size to draw more reliable conclusions in the future.

### Supplementary Information

Supplementary Information.

## Data Availability

Data supporting our findings are contained within the manuscript.

## Abbreviations

LC: Lung cancer
GWAS: Genome-wide association studies
TERT: Telomerase reverse transcriptase
CLPTM1L: Cleft lip and cleft palate transmembrane protein 1
TERC: Telomerase RNA component
SNP: Single nucleotide polymorphism
HWE: Hardy–Weinberg equilibrium
NOS: Newcastle Ottawa scale
OR: Odds ratio
95% CI: 95% Confidence interval
TSA: Trial sequential analysis
SCLC: Small cell lung carcinoma
NSCLC: Non-small-cell lung carcinoma
LUAD: Lung adenocarcinoma
LUSC: Lung squamous cell carcinoma
LCLC: Large cell lung cancer
