# Supplementary material for: Ethnicity-specific association between TERT rs2736100 (A > C) polymorphism and lung cancer risk: a comprehensive meta-analysis
Source: Sci Rep. 2023 Aug 15;13:13271. doi: 10.1038/s41598-023-40504-y (PMC10427644; doi:10.1038/s41598-023-40504-y)
Supplement: Supplementary file 1 — Supplementary Information. [file 41598_2023_40504_MOESM1_ESM.docx]

**Figure S1 Forest plot of the allele genetic model (C vs. A) for NSCLC and SCLC (Random)**

**Figure S2 Forest plot of the allele genetic model (C vs. A) for LUAD and LUSC**

**(Fixed)**

**Figure S3 Forest plot of the allele genetic model (C vs. A) for the LC smoking situation(Random)**

**Figure S4 Forest plot of the allele genetic model (C vs. A) for the NSCLC smoking situation(Random)**

**Figure S5 Forest plot of the allele genetic model (C vs. A) for the LUAD smoking situation(Random)**

Figure S6a Sensitivity analysis result of LC (C vs.A)

Figure S6b Sensitivity analysis result of LC (CC vs.AA)

Figure S6c Sensitivity analysis result of LC (CA vs.AA)

Figure S6d Sensitivity analysis result of LC (CA+CC vs.AA)

Figure S6e Sensitivity analysis result of LC (CC vs.AA+CA)

**Figure S6 Sensitivity analysis results of LC**

Figure S7a Sensitivity analysis result of various subtypes of LC (NSCLC and SCLC, C vs.A)

Figure S7b Sensitivity analysis result of various subtypes of LC (LUAD and LUSC,

C vs.A)

**Figure S7 Sensitivity analysis results of various subtypes of LC (C vs.A)**


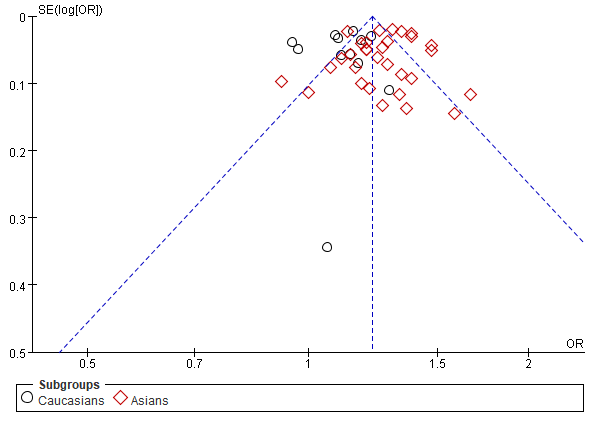


Figure S8a The funnel plot of LC (C vs.A)


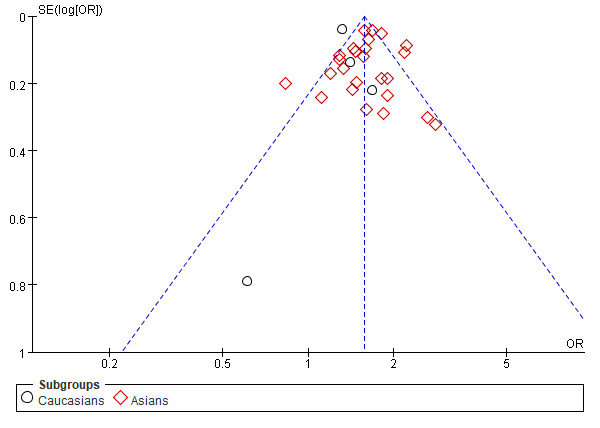


Figure S8b The funnel plot of LC (CC vs.AA)


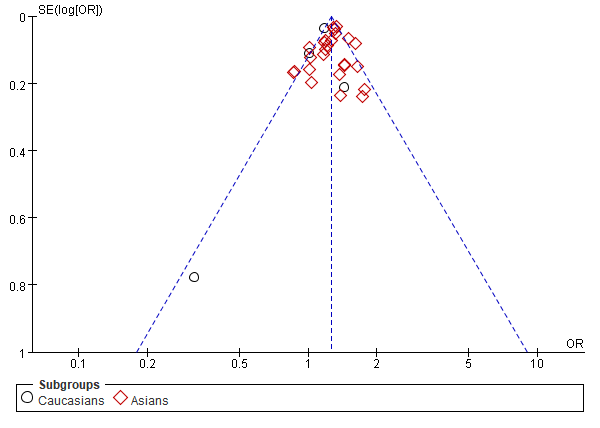


Figure S8c The funnel plot of LC (CA vs.AA)


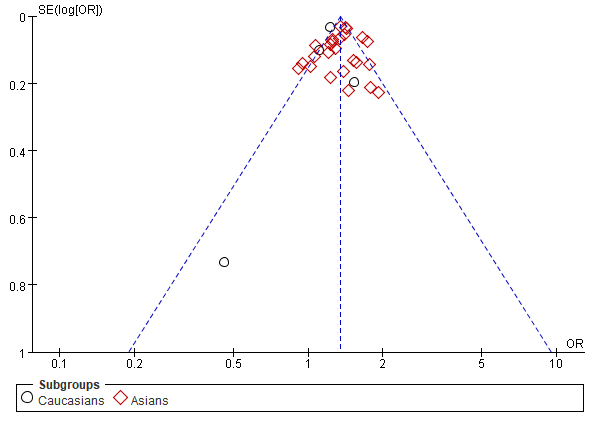


Figure S8d The funnel plot of LC (CA+CC vs.AA)


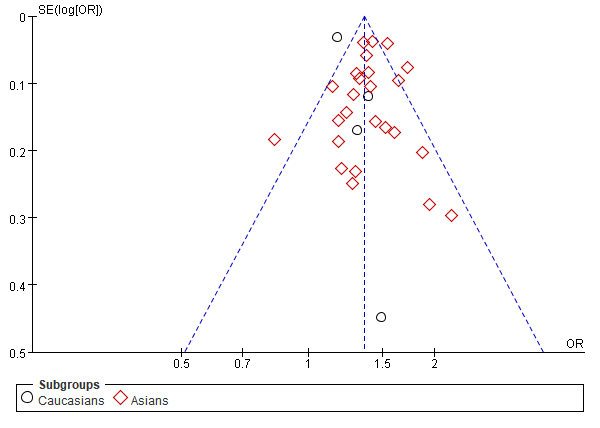


Figure S8e The funnel plot of LC (CC vs.AA+CA)

**Figure S8 The funnel plots of LC**


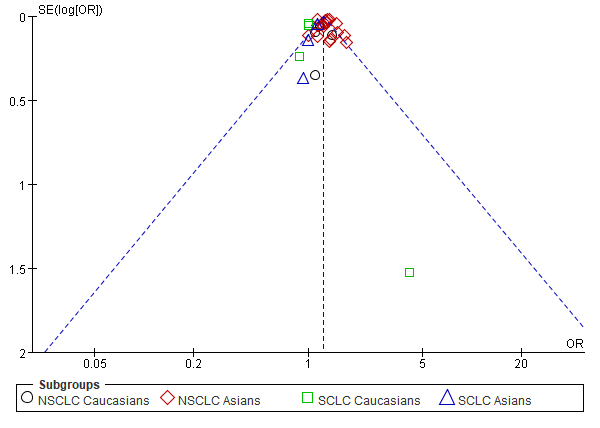


Figure S9a The funnel plot of various subtypes of LC (NSCLC and SCLC, C vs.A)


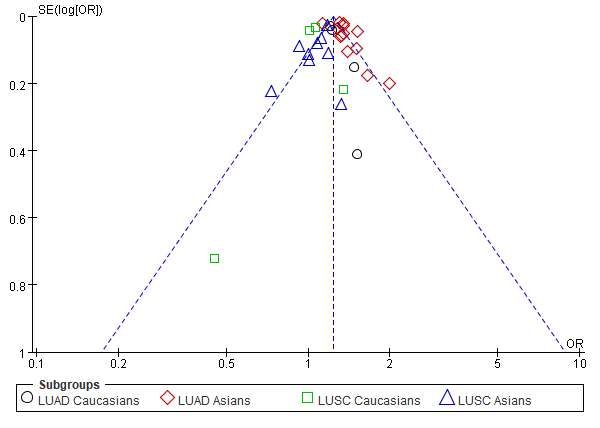


Figure S9b The funnel plot of various subtypes of LC (LUAD and LUSC, C vs.A)

**Figure S9 The funnel plots of various subtypes of LC (C vs.A)**

Figure S10a The Begg's test result of LC (C vs.A)

Figure S10b The Begg's test result of LC (CC vs.AA)

Figure S10c The Begg's test result of LC (CA vs.AA)

Figure S10d The Begg's test result of LC (CA+CC vs.AA)

Figure S10e The Begg's test result of LC (CC vs.AA+CA)

**Figure S10 The Begg's test results of LC**

Figure S11a The Egger's test result of LC (C vs.A)

Figure S11b The Egger's test result of LC (CC vs.AA)

Figure S11c The Egger's test result of LC (CA vs.AA)

Figure S11d The Egger's test result of LC (CA+CC vs.AA)

Figure S11e The Egger's test result of LC (CC vs.AA+CA)

**Figure S11 The Egger's test results of LC**

Figure S12a The Begg's test result of various subtypes of LC (NSCLC and SCLC,

C vs.A)

Figure S12b The Egger's test result of various subtypes of LC (NSCLC and SCLC,

C vs.A)

Figure S12c The Begg's test result of various subtypes of LC (LUAD and LUSC,

C vs.A)

Figure S12d The Egger's test result of various subtypes of LC (LUAD and LUSC,

C vs.A)

**Figure S12 The Begg's test and Egger's test results of various subtypes of LC**

**(C vs.A)**


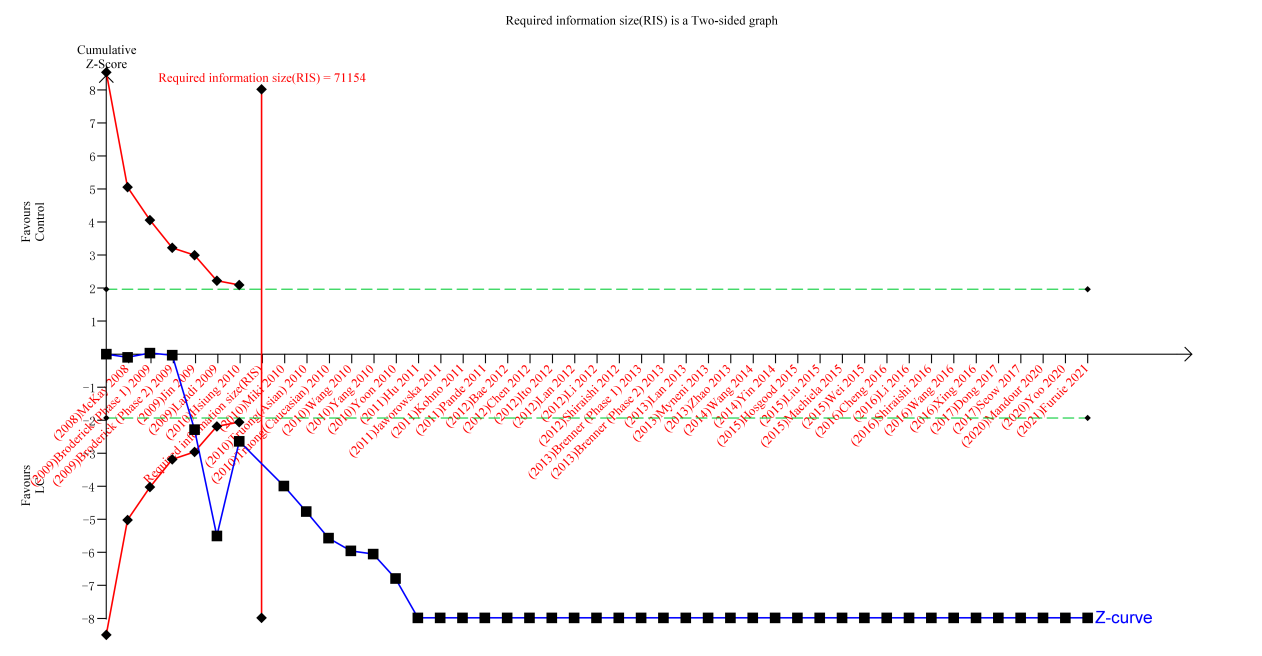


Figure S13a Trial Sequential Analysis (TSA) of LC (Overall population, C vs.A)


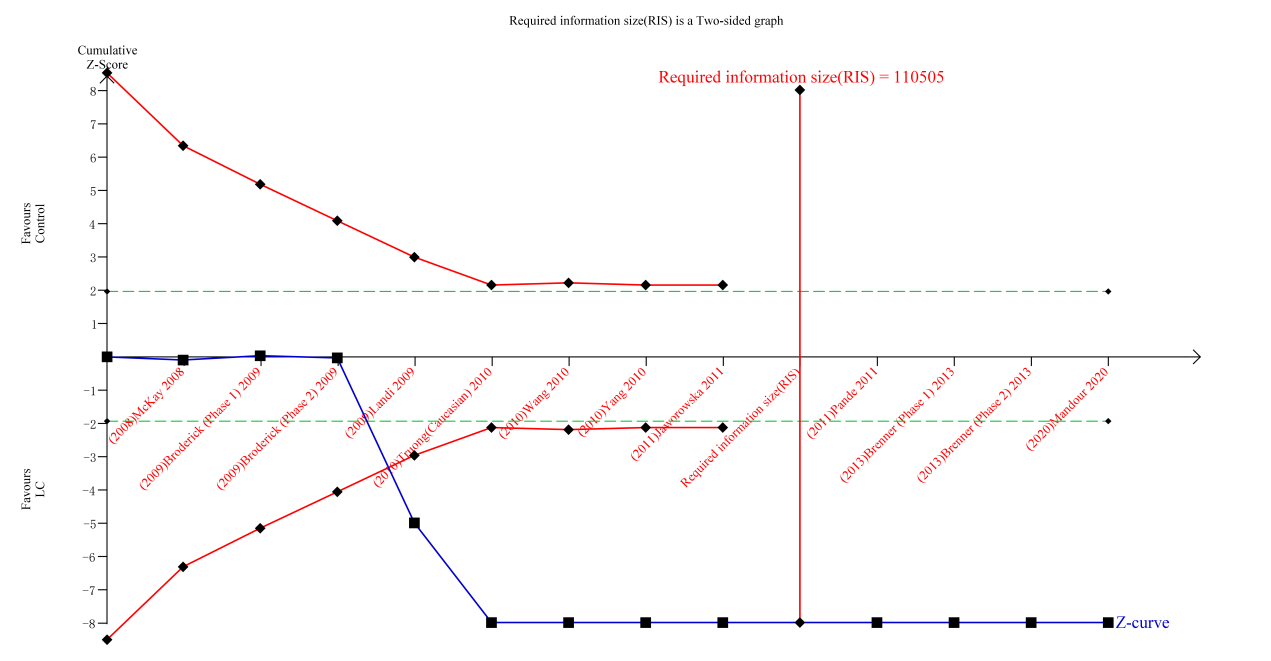


Figure S13b Trial Sequential Analysis (TSA) of LC (Caucasians, C vs.A)


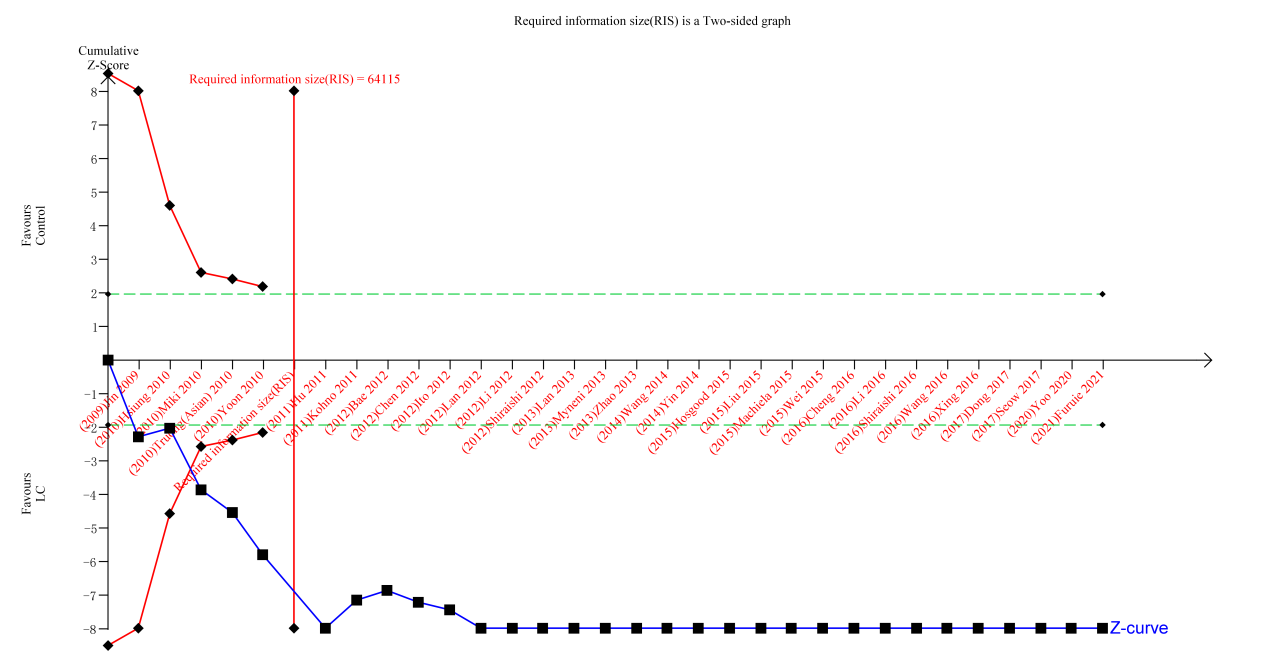


Figure S13c Trial Sequential Analysis (TSA) of LC (Asians, C vs.A)

**Figure S13 Trial Sequential Analysis (TSA) of LC (C vs.A)**


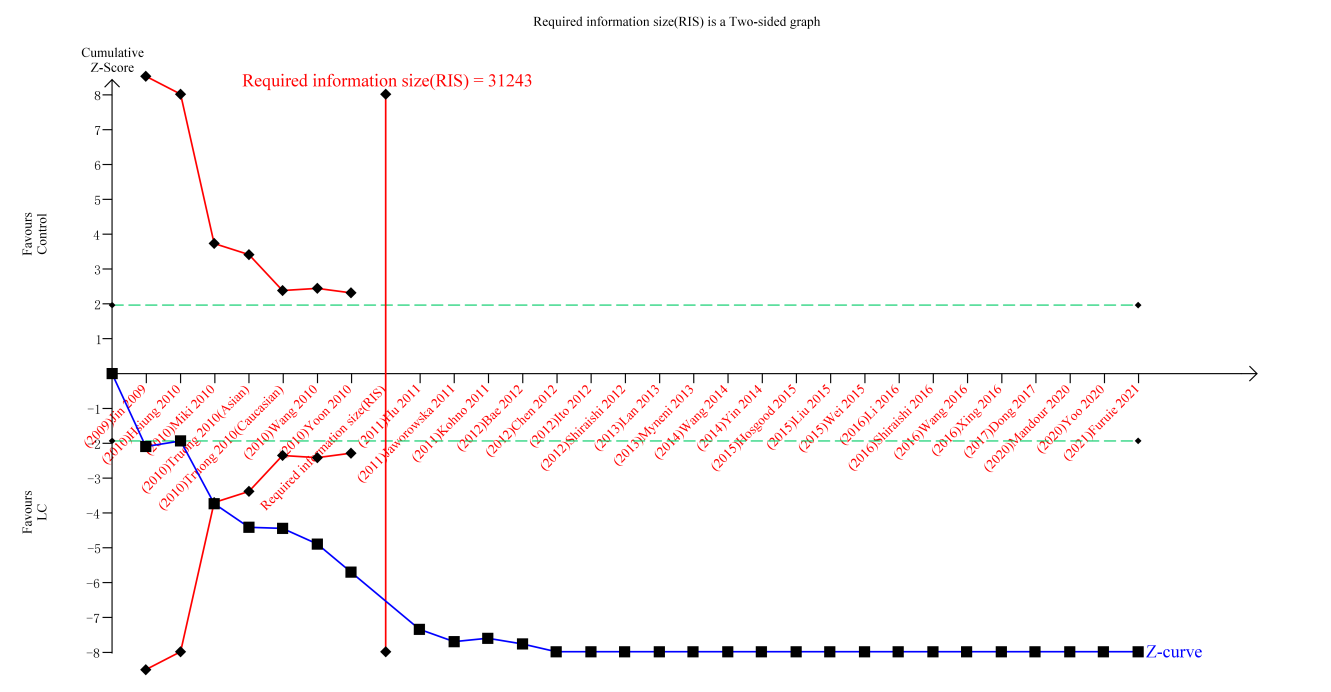


Figure S14a Trial Sequential Analysis (TSA) of LC (Overall population, CC vs.AA)


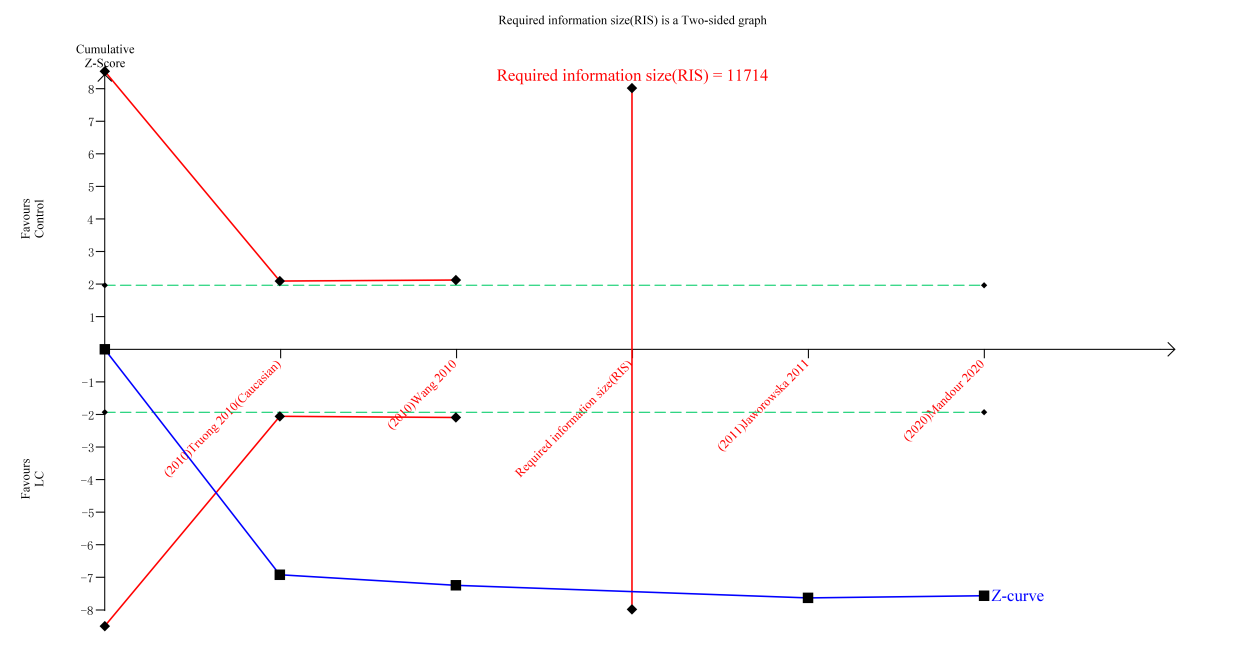


Figure S14b Trial Sequential Analysis (TSA) of LC (Caucasians, CC vs.AA)


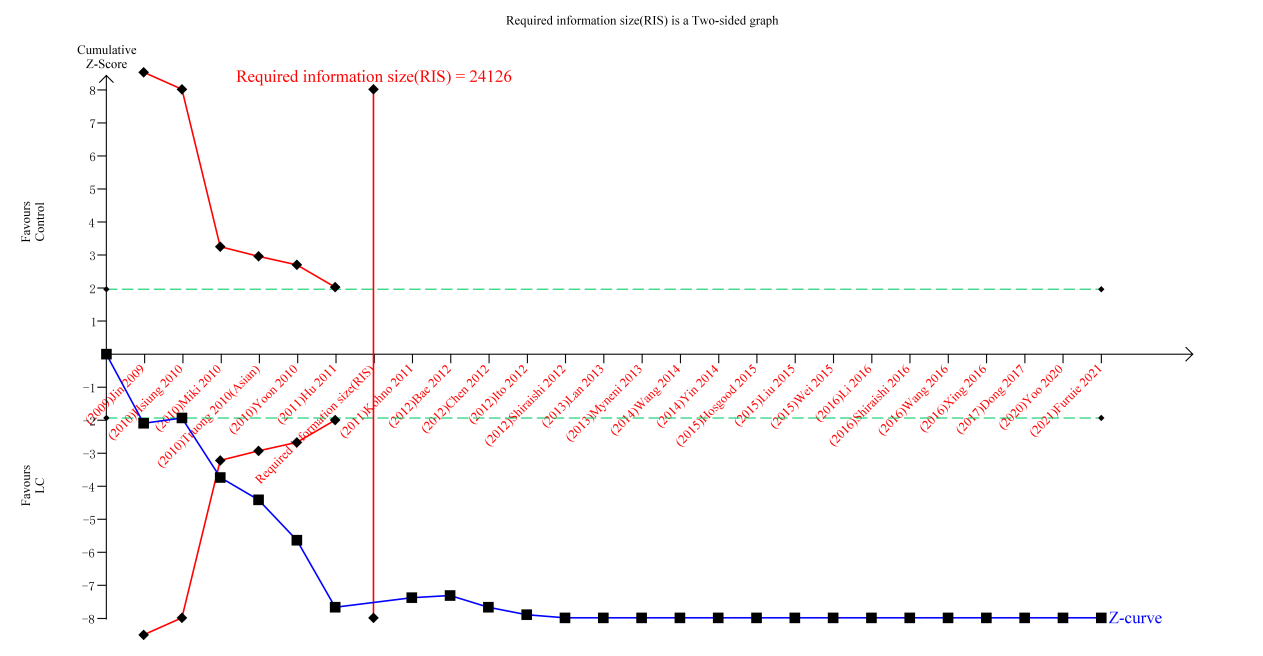


Figure S14c Trial Sequential Analysis (TSA) of LC (Asians, CC vs.AA)

**Figure S14 Trial Sequential Analysis (TSA) of LC (CC vs.AA)**


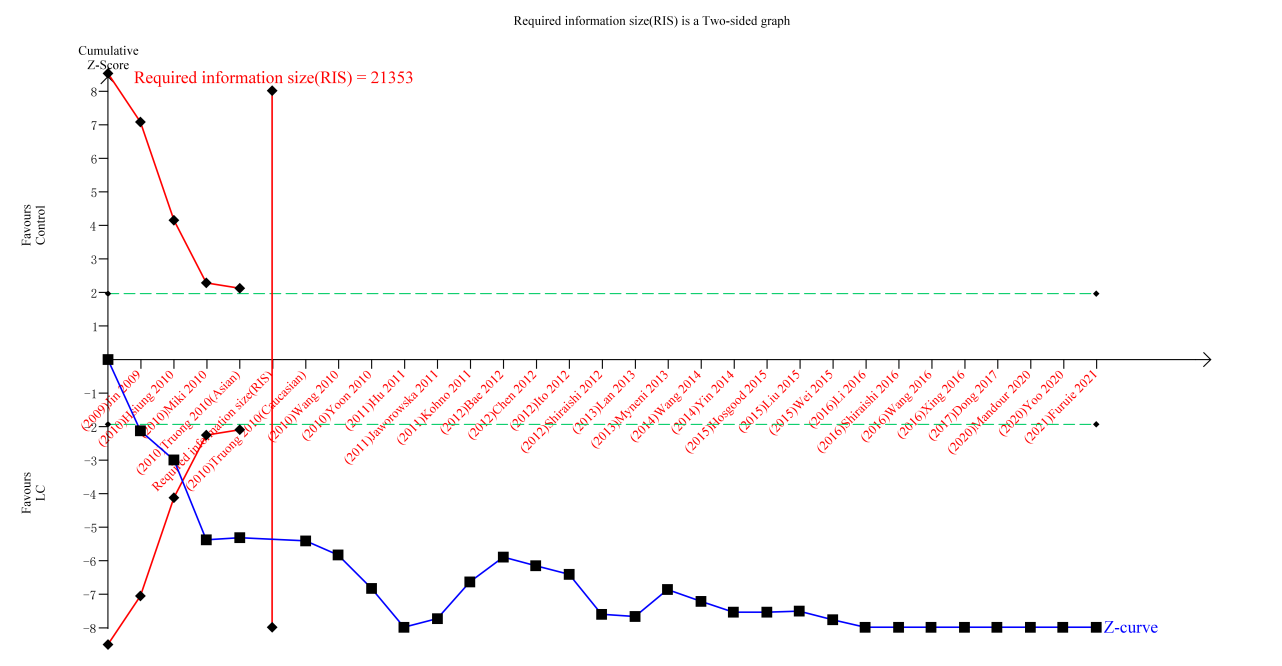


Figure S15a Trial Sequential Analysis (TSA) of LC (Overall population, CA vs.AA)


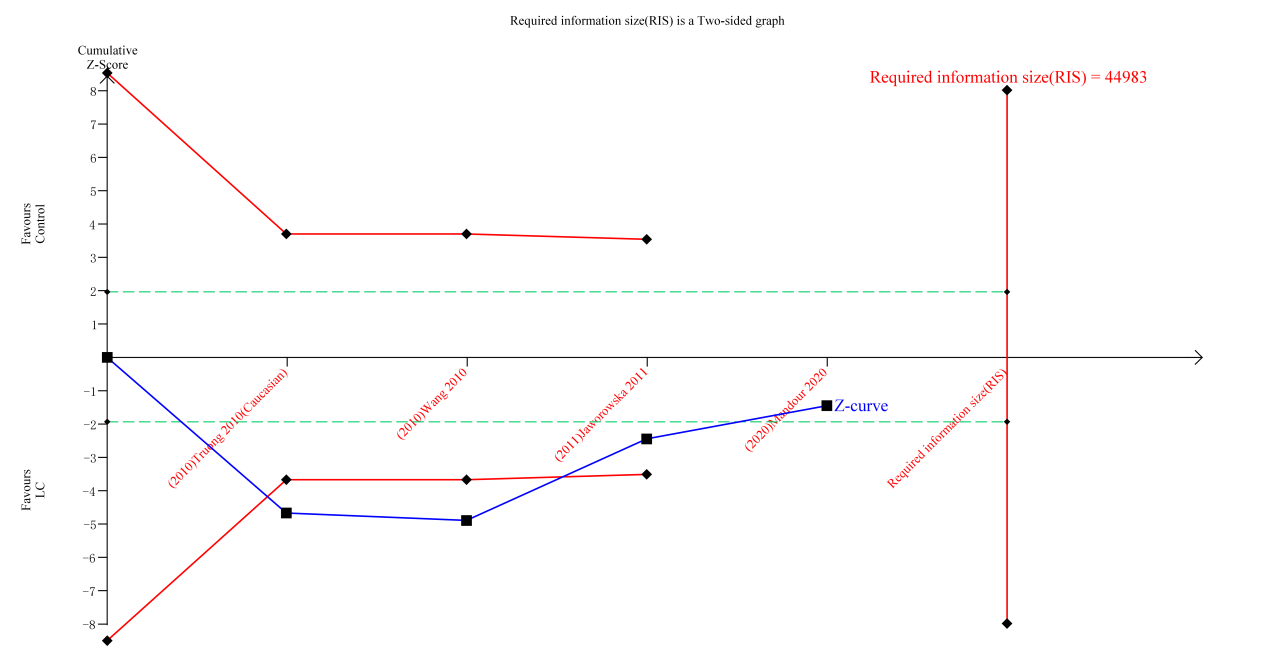


Figure S15b Trial Sequential Analysis (TSA) of LC (Caucasians, CA vs.AA)


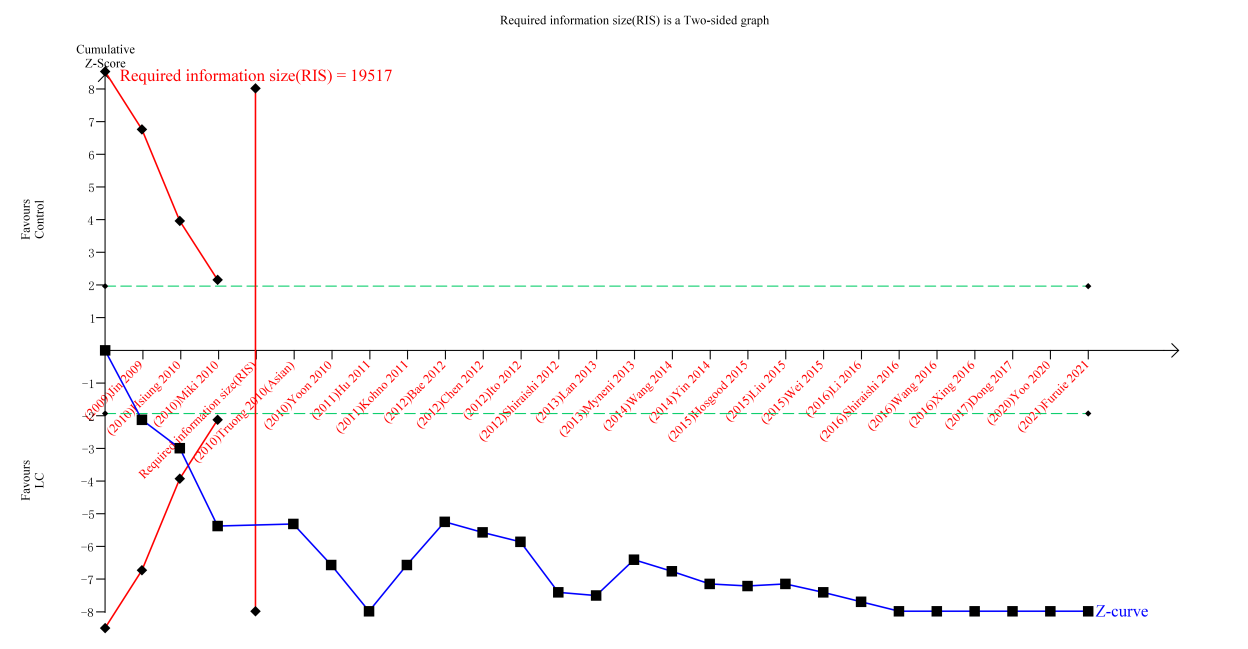


Figure S15c Trial Sequential Analysis (TSA) of LC (Asians, CA vs.AA)

**Figure S15 Trial Sequential Analysis (TSA) of LC (CA vs.AA)**


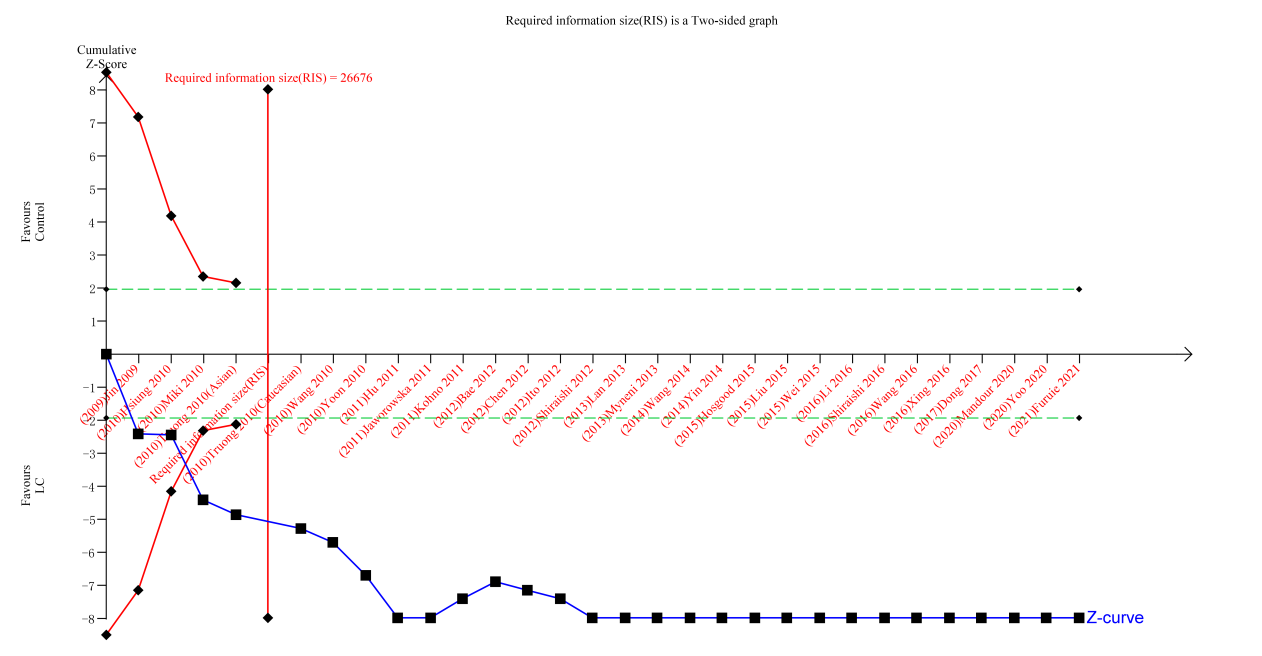


Figure S16a Trial Sequential Analysis (TSA) of LC (Overall population,

CA +CC vs.AA)


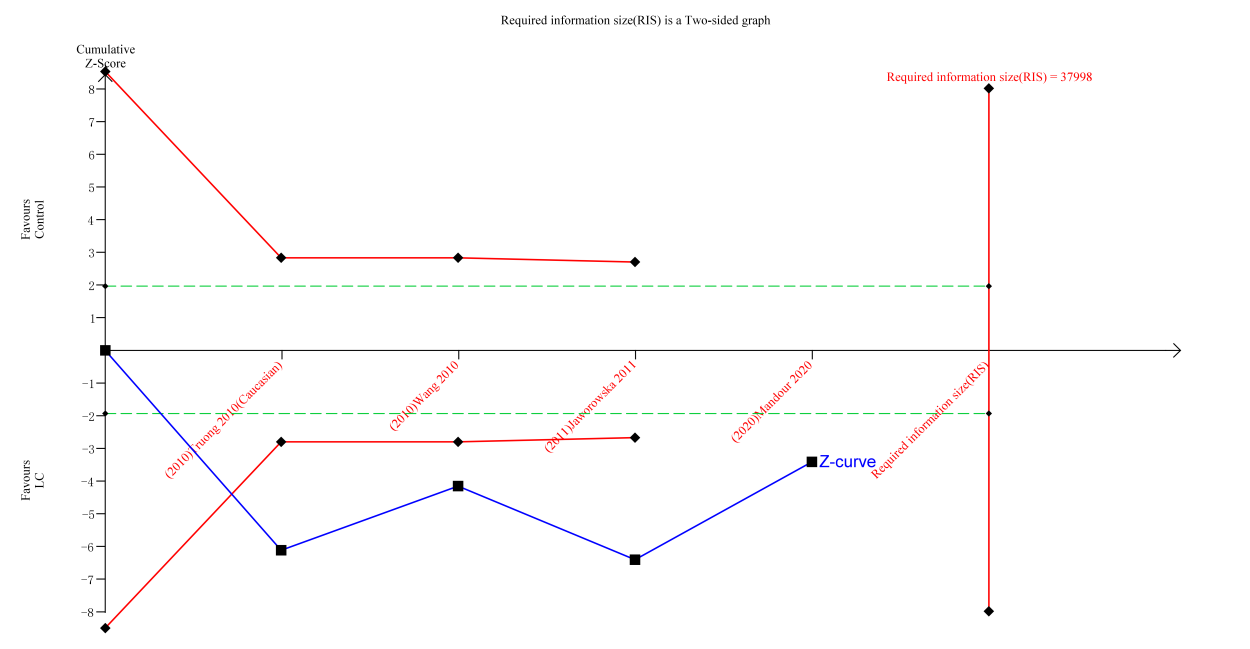


Figure S16b Trial Sequential Analysis (TSA) of LC (Caucasians,

CA +CC vs.AA)


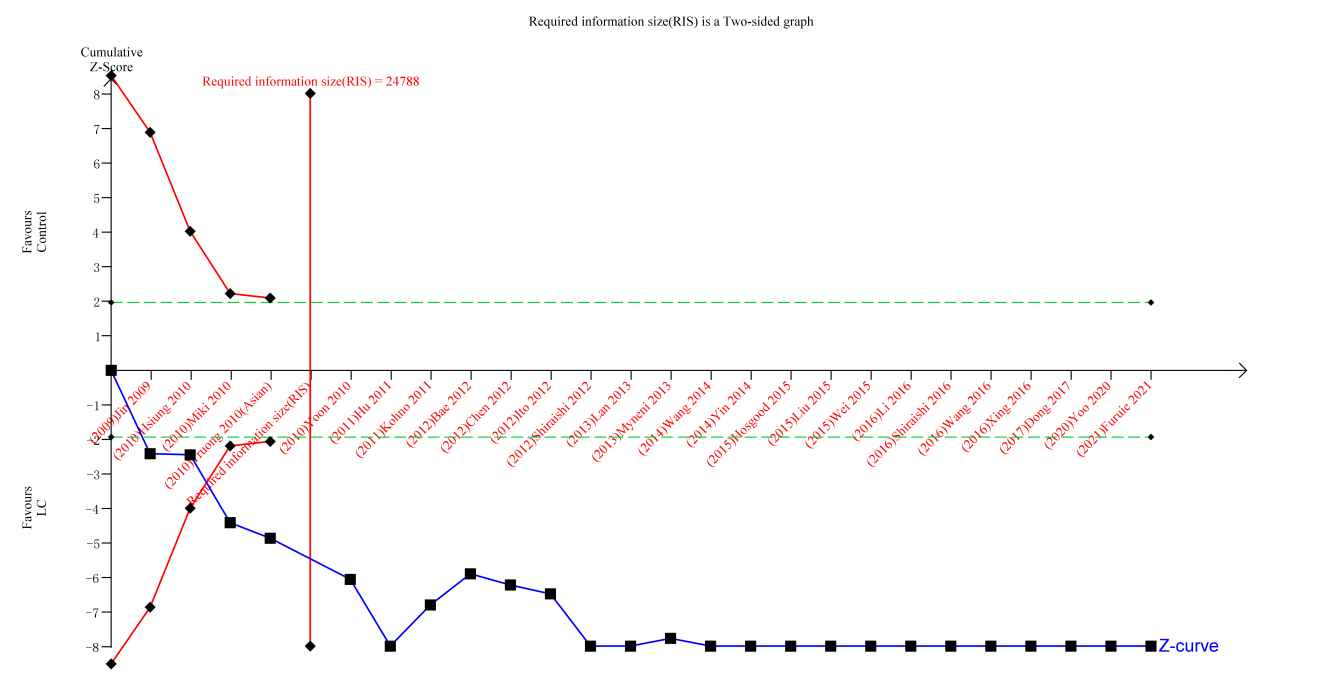


Figure S16c Trial Sequential Analysis (TSA) of LC (Asians, CA +CC vs.AA)

**Figure S16 Trial Sequential Analysis (TSA) of LC (CA +CC vs.AA)**


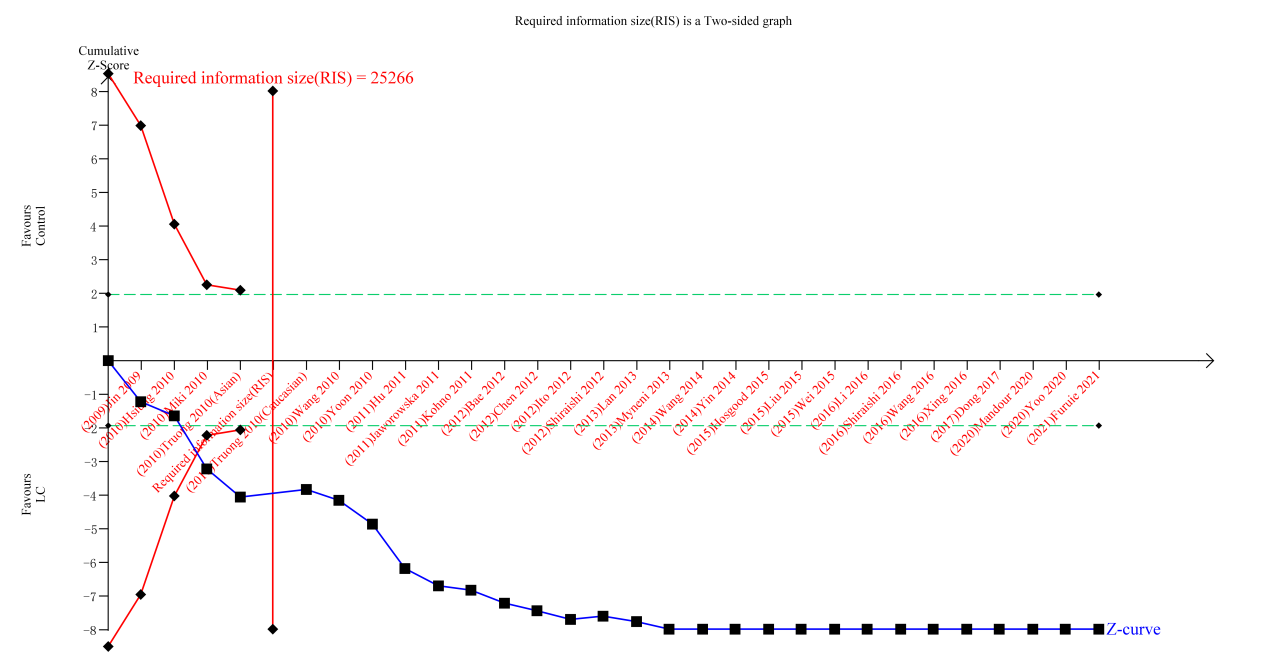


Figure S17a Trial Sequential Analysis (TSA) of LC (Overall population,

CC vs.AA+CA)


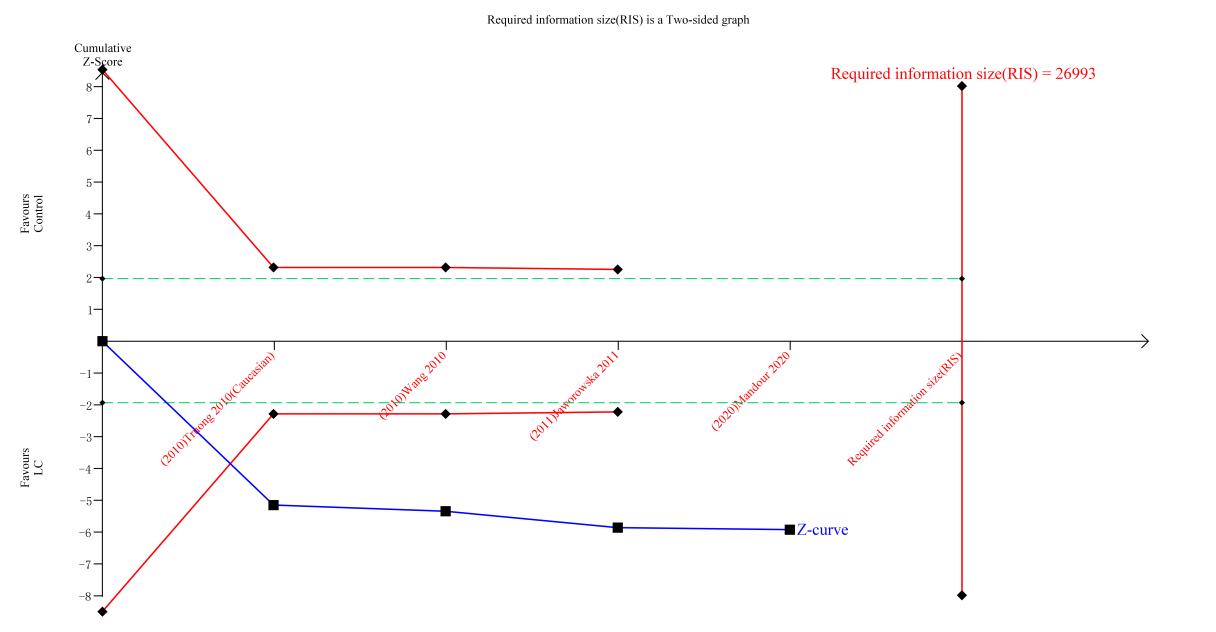


Figure S17b Trial Sequential Analysis (TSA) of LC (Caucasians,

CC vs.AA+CA)


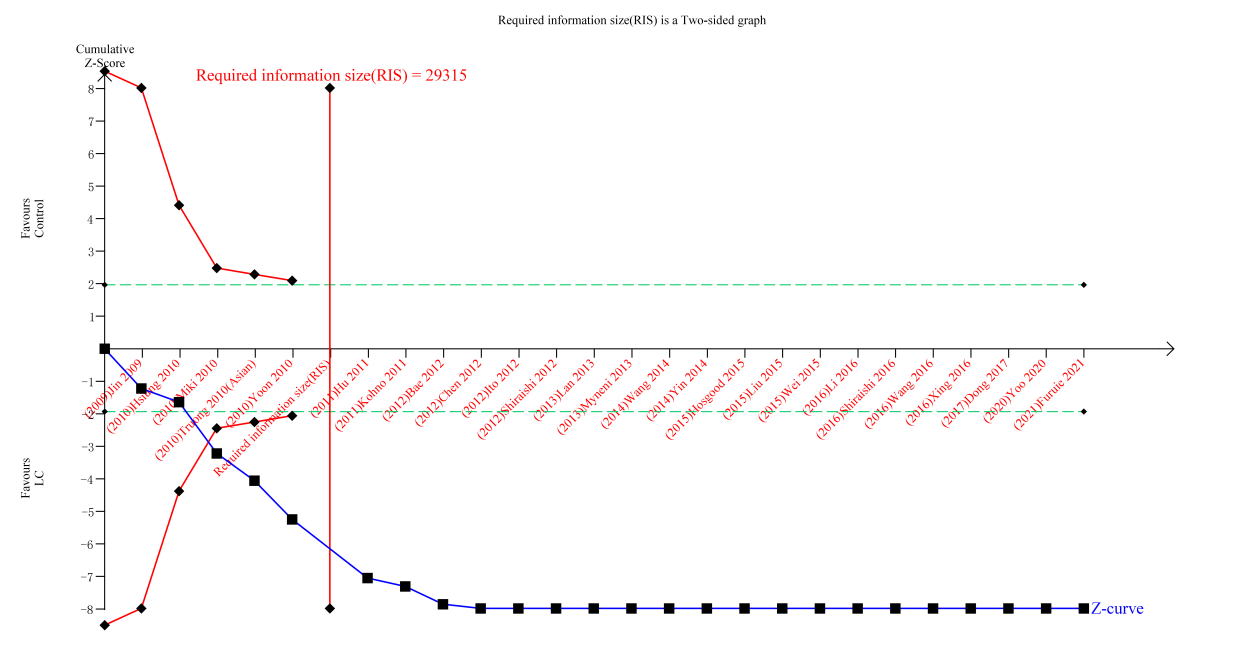


Figure S17c Trial Sequential Analysis (TSA) of LC (Asians, CC vs.AA+CA)

**Figure S17 Trial Sequential Analysis (TSA) of LC (CC vs.AA+CA)**


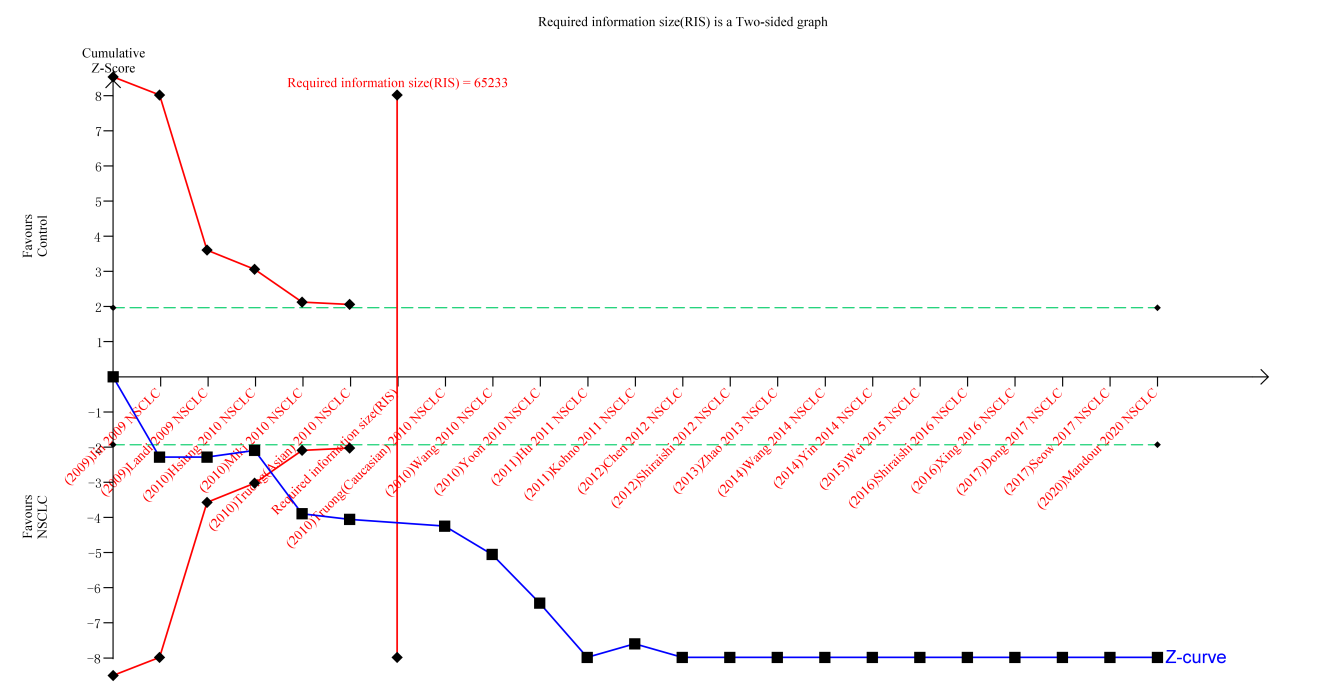


Figure S18a Trial Sequential Analysis (TSA) of NSCLC (Overall population, C vs.A)


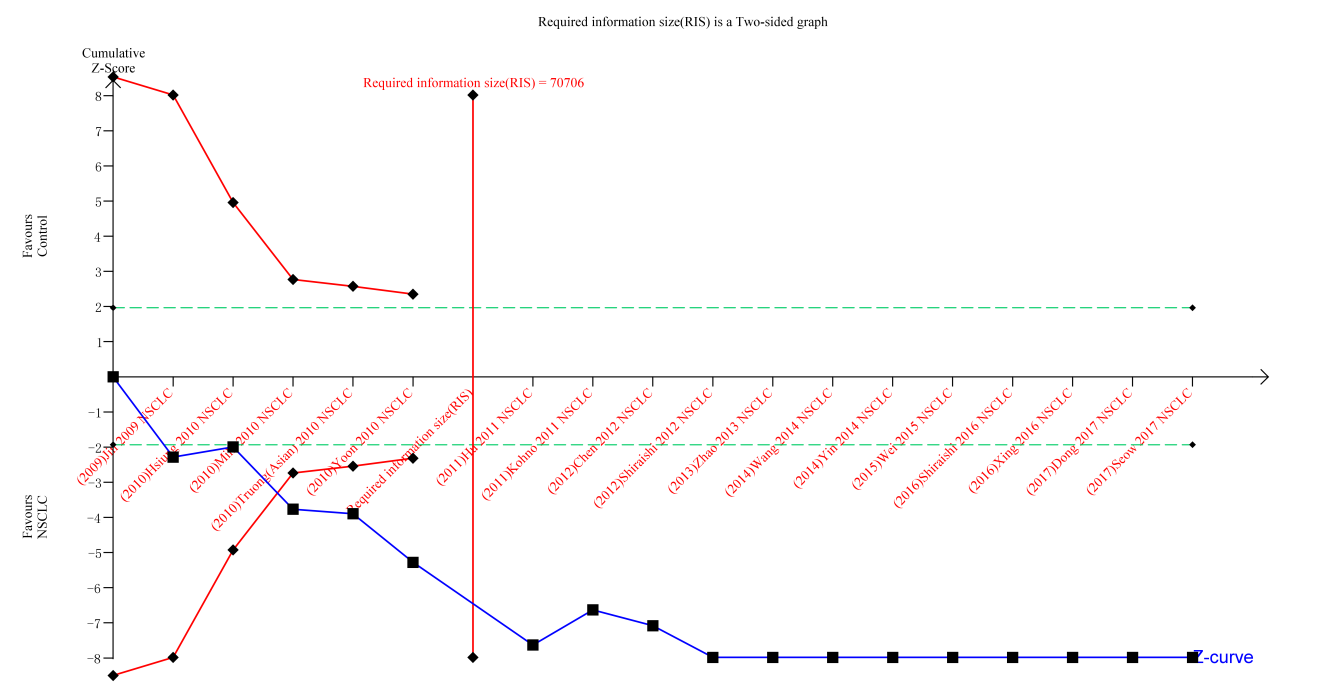


Figure S18b Trial Sequential Analysis (TSA) of NSCLC (Asians, C vs.A)

**Figure S18 Trial Sequential Analysis (TSA) of NSCLC (C vs.A)**


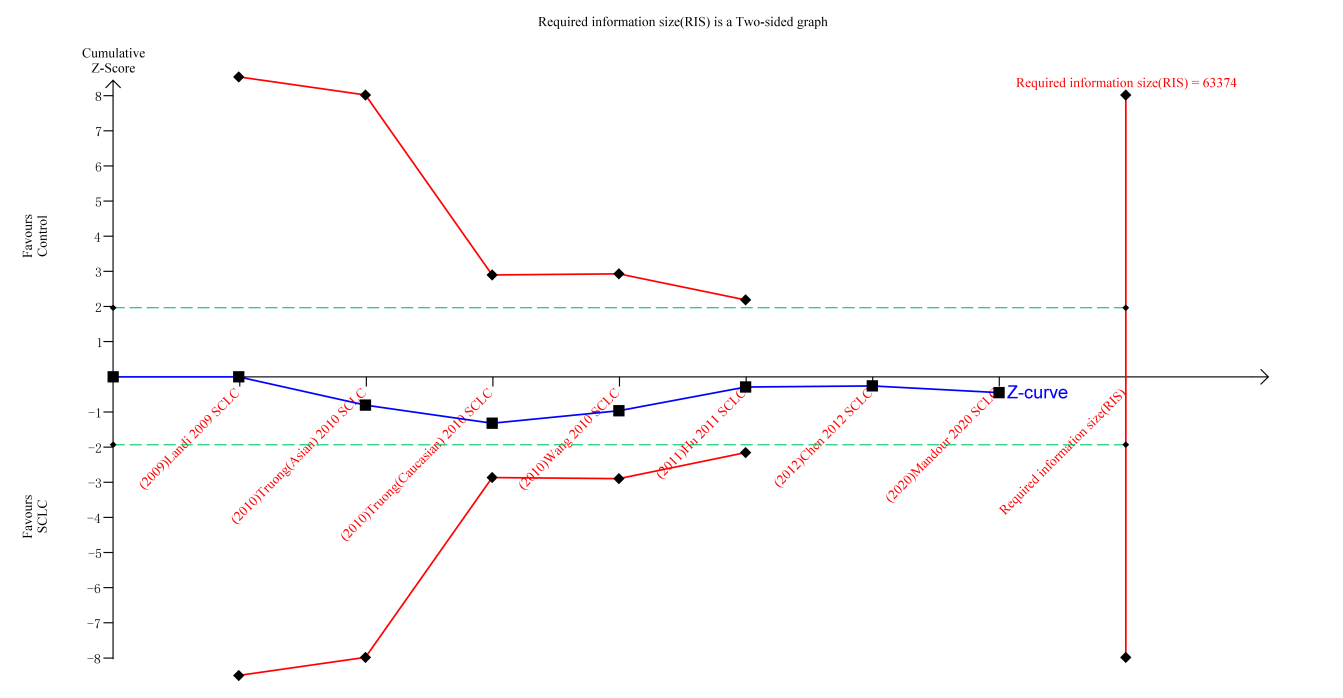


Figure S19a Trial Sequential Analysis (TSA) of SCLC (Overall population, C vs.A)


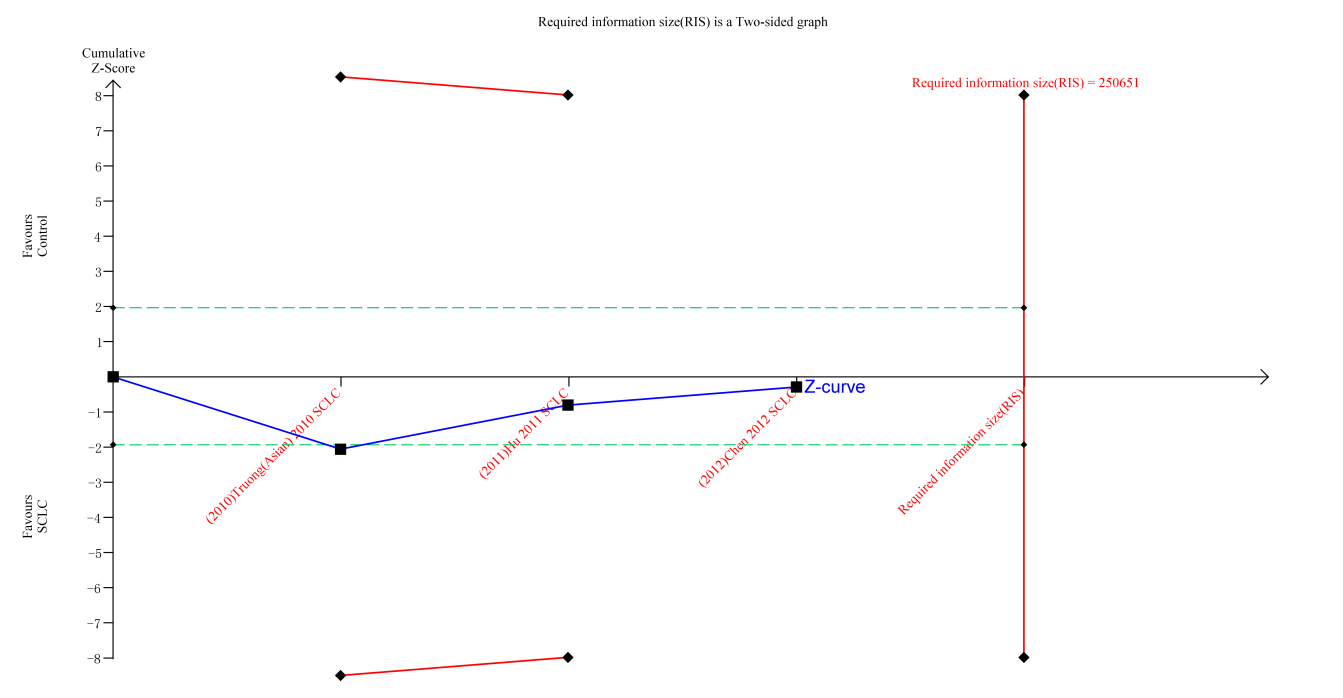


Figure S19b Trial Sequential Analysis (TSA) of SCLC (Asians, C vs.A)

**Figure S19 Trial Sequential Analysis (TSA) of SCLC (C vs.A)**


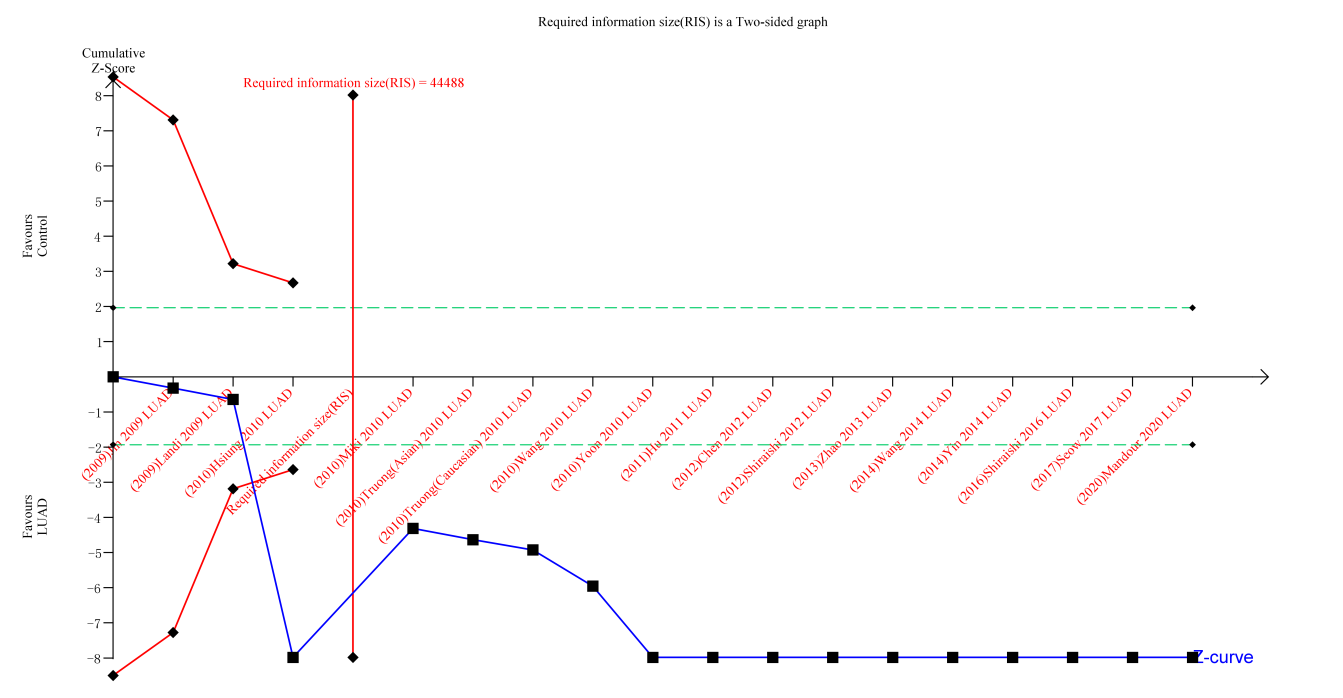


Figure S20a Trial Sequential Analysis (TSA) of LUAD (Overall population, C vs.A)


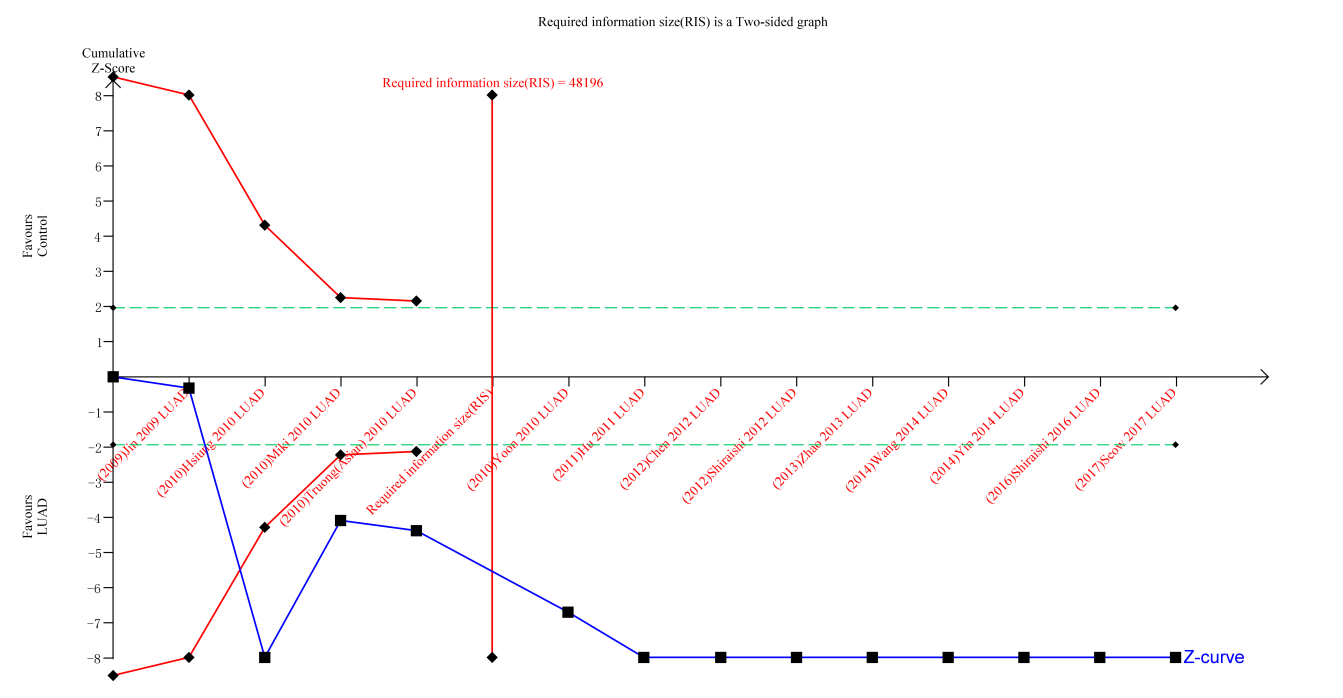


Figure S20b Trial Sequential Analysis (TSA) of LUAD (Asians, C vs.A)

**Figure S20 Trial Sequential Analysis (TSA) of LUAD (C vs.A)**


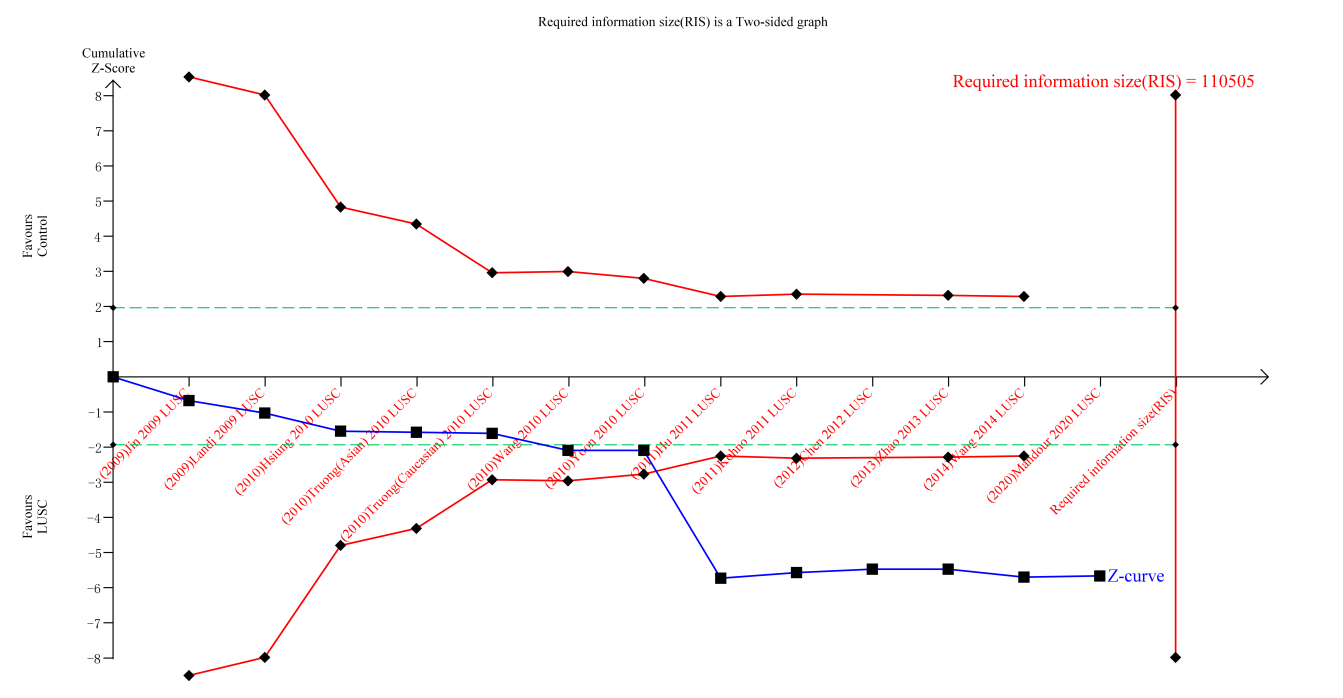


Figure S21a Trial Sequential Analysis (TSA) of LUSC (Overall population, C vs.A)


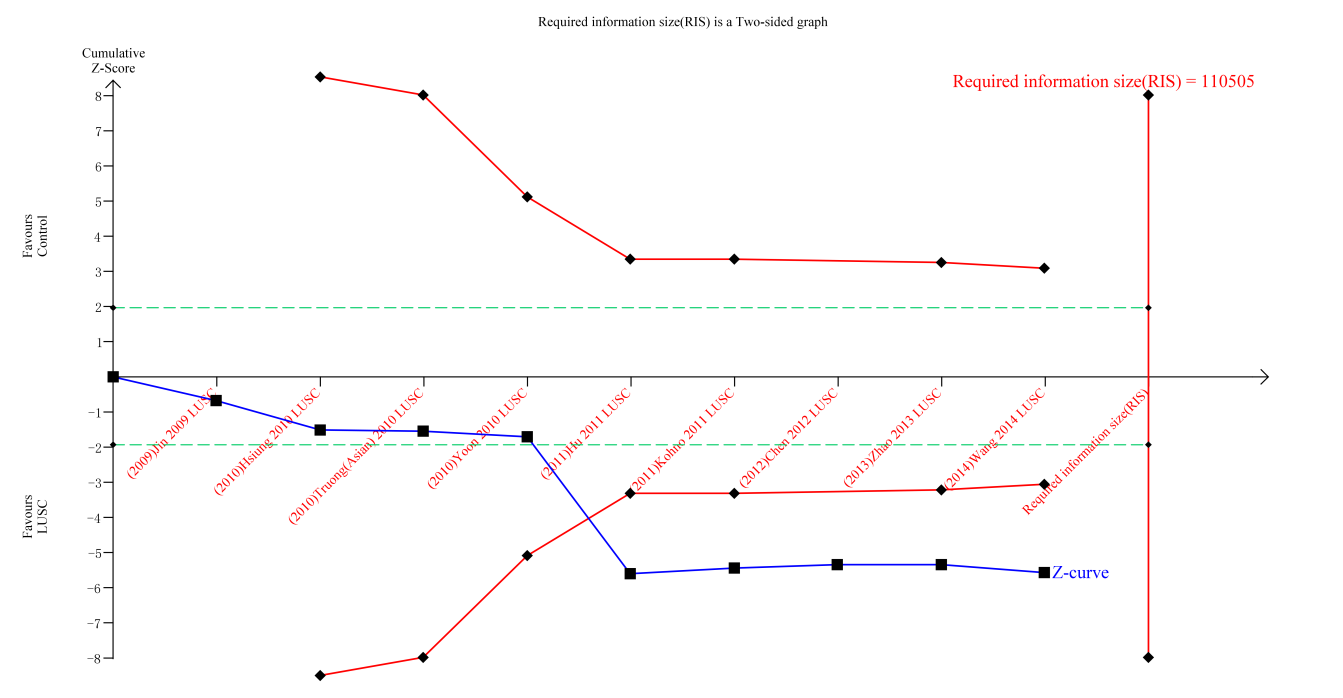


Figure S21b Trial Sequential Analysis (TSA) of LUSC (Asians, C vs.A)

**Figure S21 Trial Sequential Analysis (TSA) of LUSC (C vs.A)**


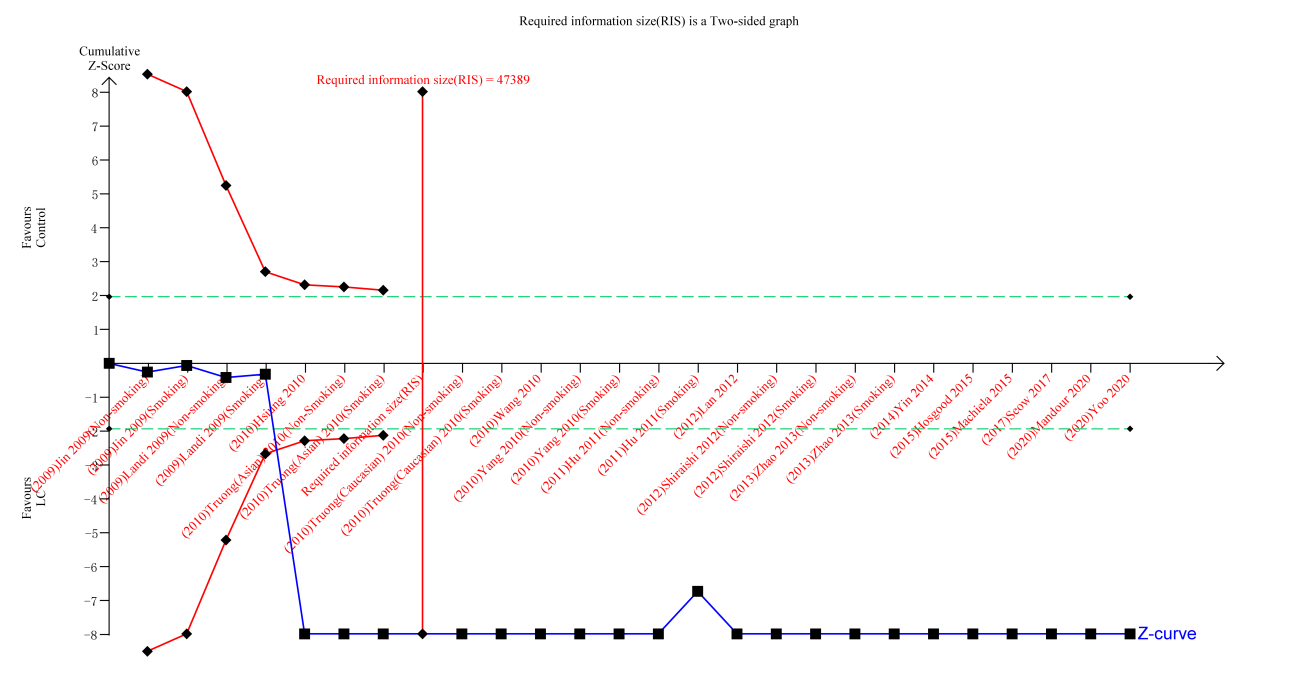


Figure S22a Trial Sequential Analysis (TSA) of LC smoking status (Total Smoking and Non-smoking, C vs.A)


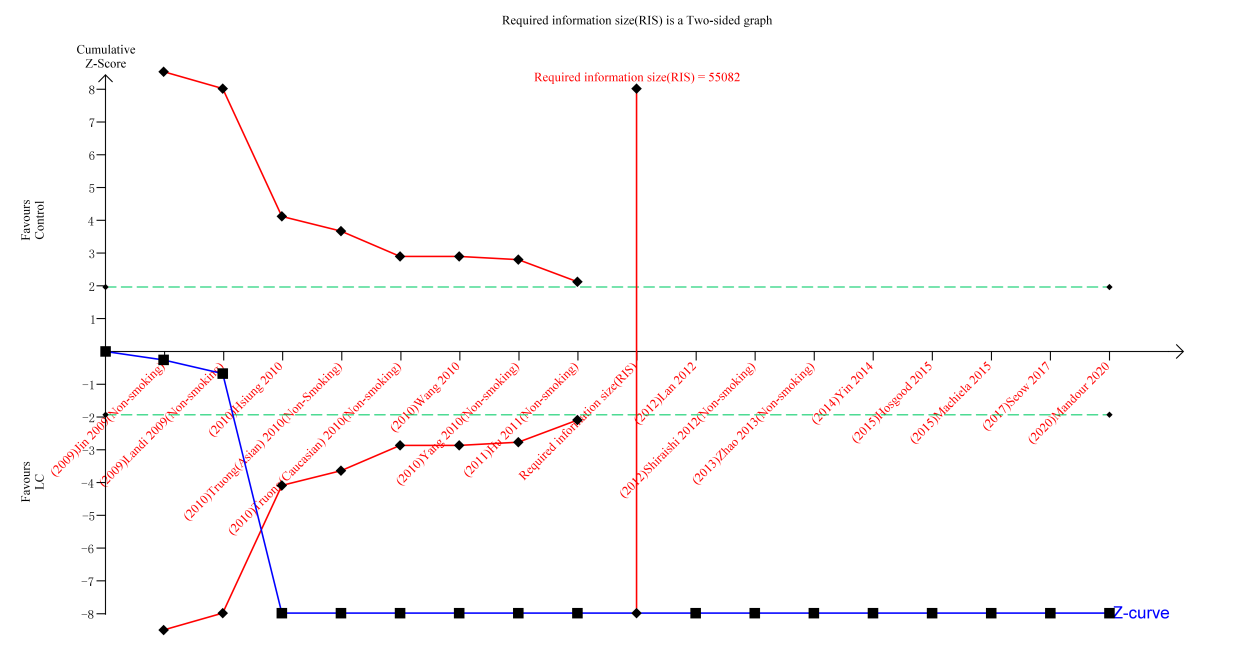


Figure S22b Trial Sequential Analysis (TSA) of LC smoking status (Total Non-smoking, C vs.A)


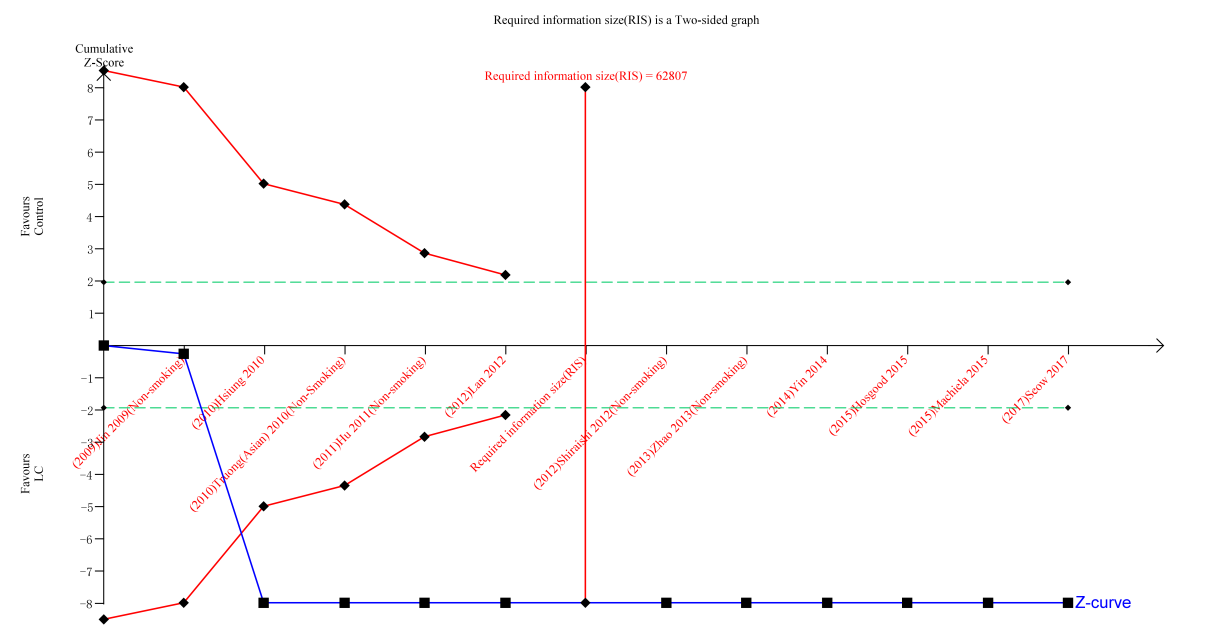


Figure S22c Trial Sequential Analysis (TSA) of LC smoking status (Asians Non-smoking, C vs.A)

**Figure S22 Trial Sequential Analysis (TSA) of LC smoking status (C vs.A)**


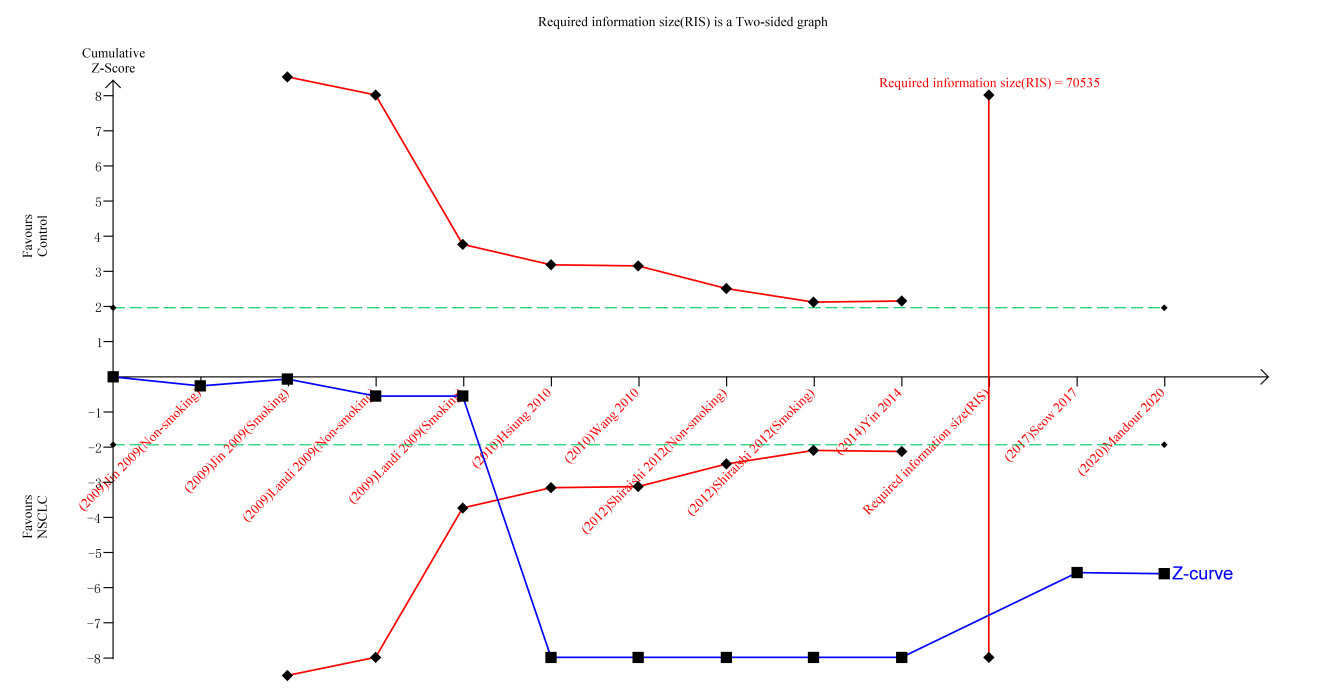


Figure S23a Trial Sequential Analysis (TSA) of NSCLC smoking status (Total Smoking and Non-smoking, C vs.A)


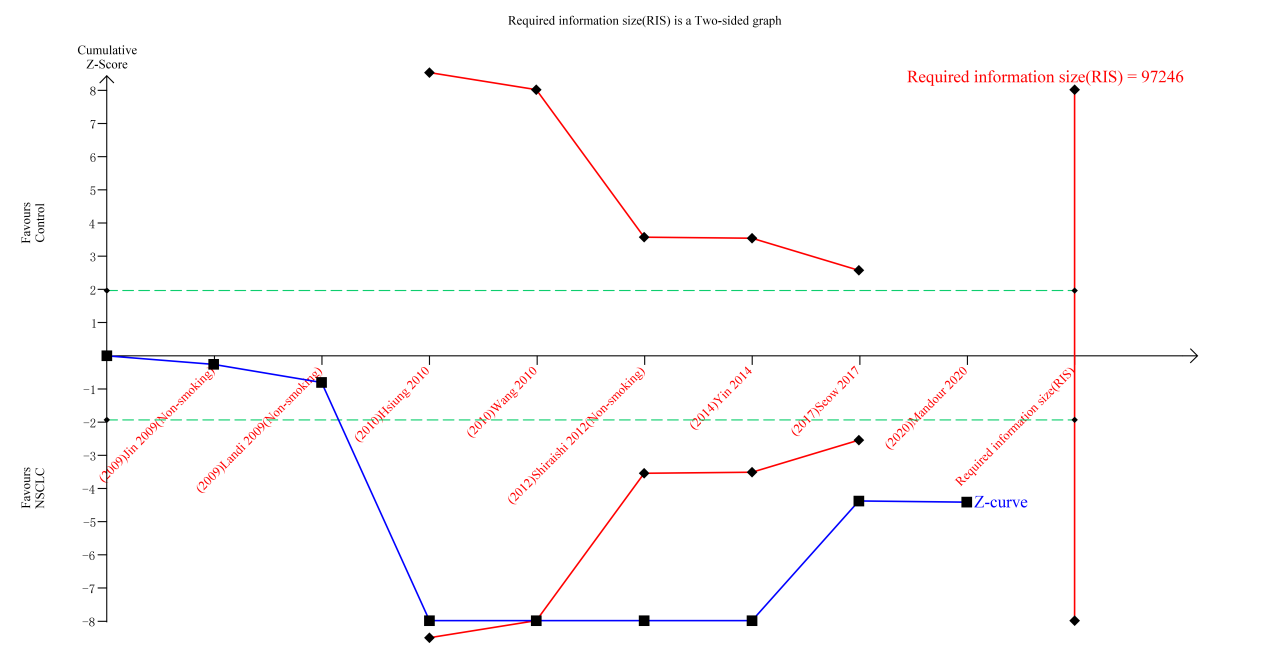


Figure S23b Trial Sequential Analysis (TSA) of NSCLC smoking status (Total Non-smoking, C vs.A)


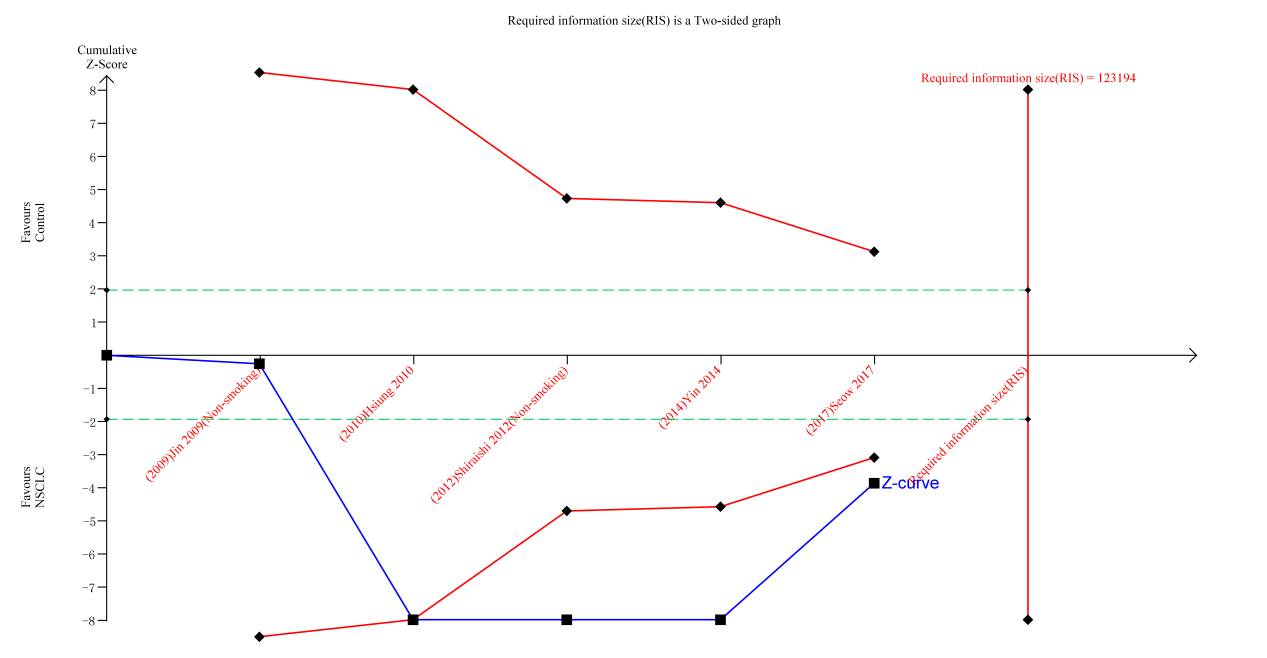


Figure S23c Trial Sequential Analysis (TSA) of NSCLC smoking status (Asians Non-smoking, C vs.A)

**Figure S23 Trial Sequential Analysis (TSA) of NSCLC smoking status (C vs.A)**


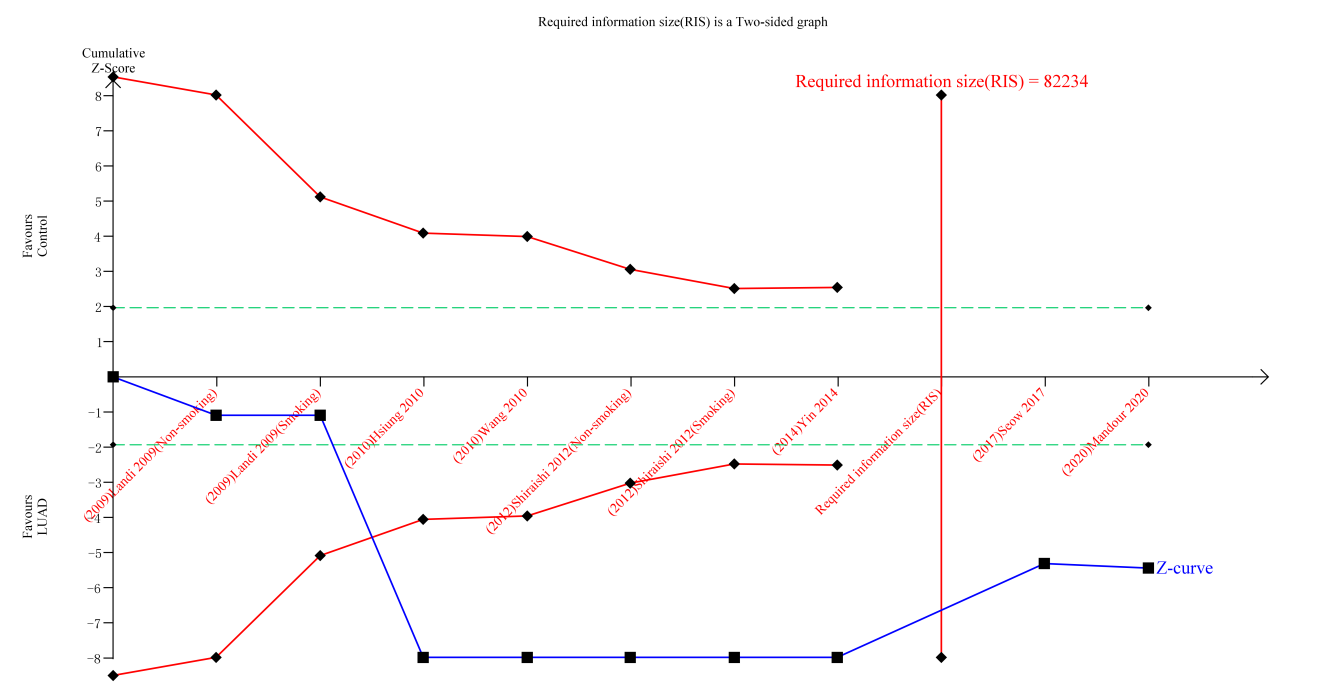


Figure S24a Trial Sequential Analysis (TSA) of LUAD smoking status (Total Smoking and Non-smoking, C vs.A)


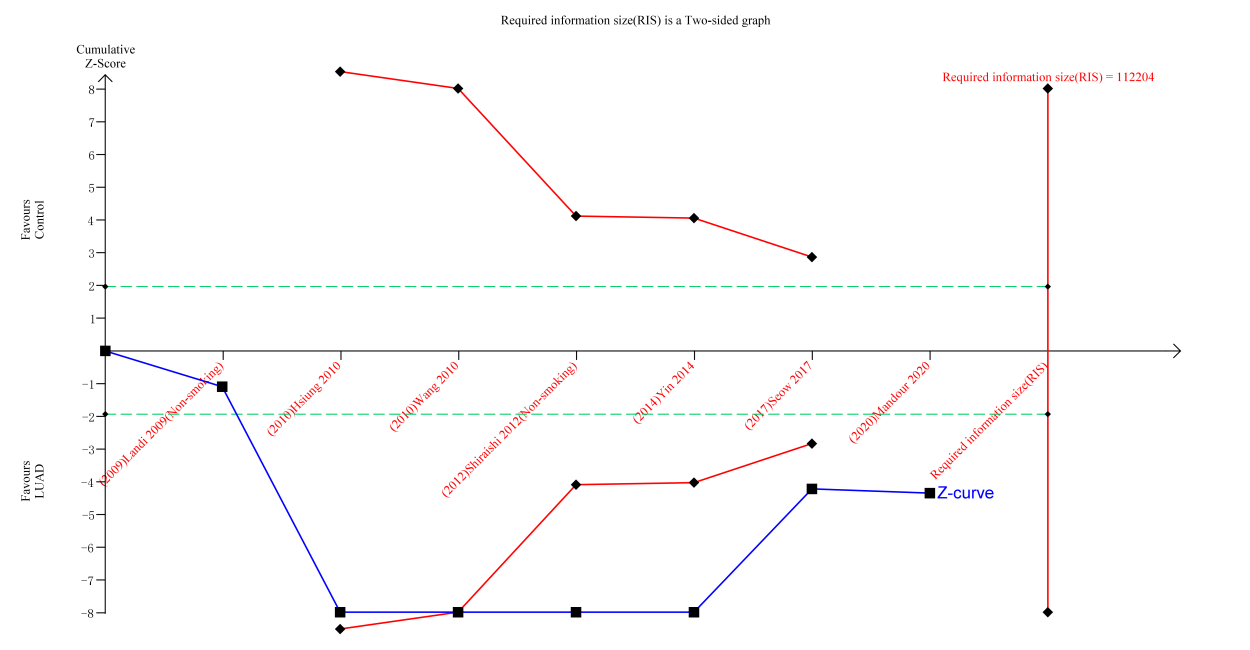


Figure S24b Trial Sequential Analysis (TSA) of LUAD smoking status (Total Non-smoking, C vs.A)


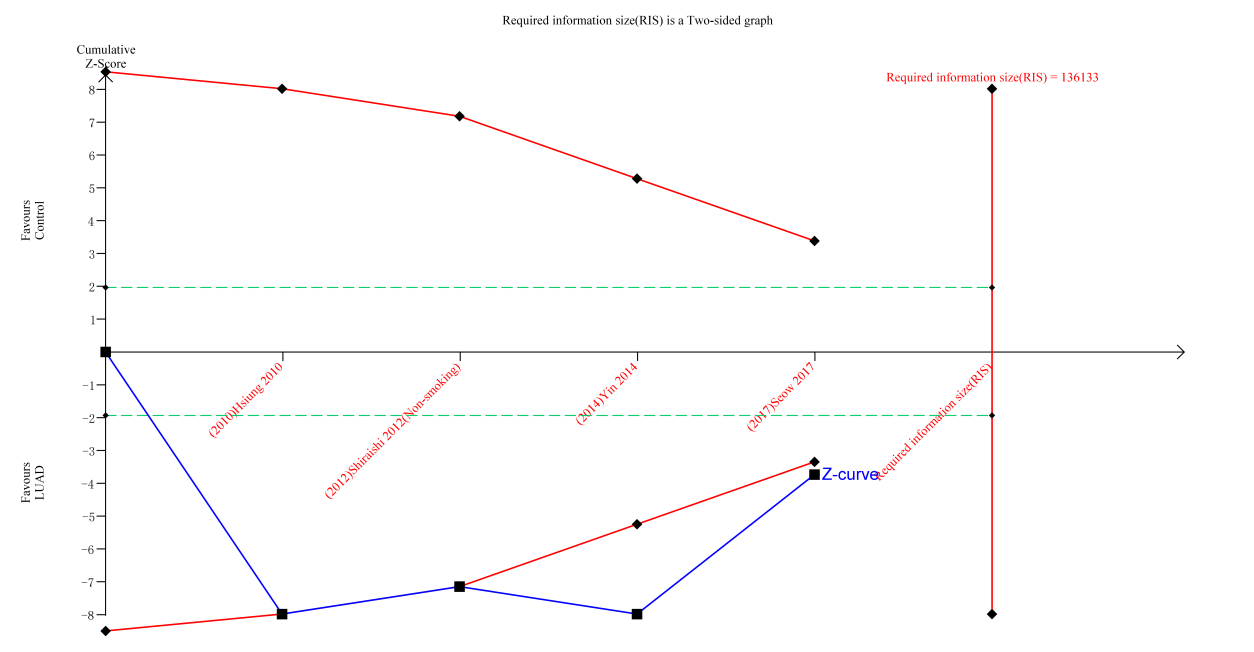


Figure S24c Trial Sequential Analysis (TSA) of LUAD smoking status (Asians Non-smoking, C vs.A)

**Figure S24 Trial Sequential Analysis (TSA) of LUAD smoking status (C vs.A)**

**Table S1 PubMed search strategy**

| Search number | Query | Search Details | Results |
| --- | --- | --- | --- |
| #4 | ((#1) AND (#2)) AND (#3) | ("lung neoplasms"[MeSH Terms] OR "LC"[Title/Abstract] OR "lung cancer"[Title/Abstract]) AND ("Telomerase"[MeSH Terms] OR "TERT"[Title/Abstract] OR "telomerase reverse transcriptase"[Title/Abstract] OR "rs2736100"[Title/Abstract]) AND "polymorphism, genetic"[MeSH Terms] | 104 |
| #3 | polymorphism[MeSH Terms] | "polymorphism, genetic"[MeSH Terms] | 298,342 |
| #2 | (((Telomerase reverse transcriptase[MeSH Terms]) OR (TERT[Title/Abstract])) OR (Telomerase reverse transcriptase[Title/Abstract])) OR (rs2736100[Title/Abstract]) | "Telomerase"[MeSH Terms] OR "TERT"[Title/Abstract] OR "telomerase reverse transcriptase"[Title/Abstract] OR "rs2736100"[Title/Abstract] | 43,486 |
| #1 | ((Lung cancer[MeSH Terms]) OR (LC[Title/Abstract])) OR (Lung cancer[Title/Abstract]) | "lung neoplasms"[MeSH Terms] OR "LC"[Title/Abstract] OR "lung cancer"[Title/Abstract] | 438,064 |

**Table S2 Sensitivity analysis results of C vs.A**

| Study omitted | Year | Estimate | [95% Conf. Interval] | |
| --- | --- | --- | --- | --- |
| Bae | 2012 | 0.19379424 | 0.15999822 | 0.22759026 |
| Brenner (Phase 1) | 2013 | 0.19155519 | 0.15701003 | 0.22610036 |
| Brenner (Phase 2) | 2013 | 0.19472185 | 0.16094957 | 0.22849414 |
| Broderick (Phase 1) | 2009 | 0.19767754 | 0.16500382 | 0.23035127 |
| Broderick (Phase 2) | 2009 | 0.19890538 | 0.16746217 | 0.23034857 |
| Chen | 2012 | 0.19127974 | 0.15766047 | 0.22489901 |
| Cheng | 2016 | 0.19203366 | 0.15779595 | 0.22627138 |
| Dong | 2017 | 0.19046691 | 0.15689121 | 0.22404262 |
| Furuie | 2021 | 0.19614159 | 0.16294022 | 0.22934294 |
| Hosgood | 2015 | 0.18642914 | 0.15322734 | 0.21963094 |
| Hsiung | 2010 | 0.18599927 | 0.15303501 | 0.21896353 |
| Hu | 2011 | 0.19080392 | 0.15567973 | 0.22592808 |
| Ito | 2012 | 0.19263101 | 0.15880959 | 0.22645241 |
| Jaworowska | 2011 | 0.19249958 | 0.15863425 | 0.2263649 |
| Jin | 2009 | 0.19327608 | 0.1593667 | 0.22718544 |
| Kohno | 2011 | 0.19432512 | 0.16084151 | 0.22780873 |
| Lan | 2013 | 0.18906203 | 0.15566413 | 0.22245994 |
| Lan | 2012 | 0.18733034 | 0.15409949 | 0.22056118 |
| Landi | 2009 | 0.19509904 | 0.16154975 | 0.22864832 |
| Li | 2012 | 0.19252394 | 0.15833439 | 0.22671349 |
| Li | 2016 | 0.19173895 | 0.15804093 | 0.22543696 |
| Liu | 2015 | 0.1904593 | 0.15682286 | 0.22409575 |
| Machiela | 2015 | 0.18747354 | 0.15405071 | 0.22089636 |
| Mandour | 2020 | 0.19202299 | 0.15858501 | 0.22546098 |
| McKay | 2008 | 0.19256097 | 0.15827234 | 0.22684959 |
| Miki | 2010 | 0.19007455 | 0.15587176 | 0.22427735 |
| Myneni | 2013 | 0.19214229 | 0.15842228 | 0.22586231 |
| Pande | 2011 | 0.19327608 | 0.1593667 | 0.22718544 |
| Seow | 2017 | 0.19405547 | 0.15986642 | 0.22824451 |
| Shiraishi | 2016 | 0.18941097 | 0.15482962 | 0.22399232 |
| Shiraishi | 2012 | 0.18832871 | 0.15451096 | 0.22214648 |
| Truong(Asian) | 2010 | 0.19199976 | 0.15791573 | 0.2260838 |
| Truong(Caucasian) | 2010 | 0.19350168 | 0.15897138 | 0.22803196 |
| Wang | 2014 | 0.19199976 | 0.15791573 | 0.2260838 |
| Wang | 2016 | 0.18946232 | 0.15581551 | 0.22310914 |
| Wang | 2010 | 0.19081755 | 0.15714312 | 0.224492 |
| Wei | 2015 | 0.1911616 | 0.15720588 | 0.22511733 |
| Xing | 2016 | 0.18753339 | 0.1543048 | 0.220762 |
| Yang | 2010 | 0.19390795 | 0.16009183 | 0.22772405 |
| Yin | 2014 | 0.18985321 | 0.15614016 | 0.22356626 |
| Yoo | 2020 | 0.194258 | 0.16060205 | 0.22791398 |
| Yoon | 2010 | 0.19063576 | 0.15653831 | 0.2247332 |
| Zhao | 2013 | 0.19053297 | 0.15669048 | 0.22437547 |
| Combined |  | 0.19171104 | 0.15836752 | 0.22505456 |

**Table S3 Sensitivity analysis results of CC vs.AA**

| Study omitted | Year | Estimate | [95% Conf. Interval] | |
| --- | --- | --- | --- | --- |
| Bae | 2012 | 0.45541438 | 0.38091335 | 0.52991539 |
| Chen | 2012 | 0.44732356 | 0.37320954 | 0.52143759 |
| Dong | 2017 | 0.44550845 | 0.37154999 | 0.51946694 |
| Furuie | 2021 | 0.46277466 | 0.39241776 | 0.5331316 |
| Hosgood | 2015 | 0.43293211 | 0.36098197 | 0.50488228 |
| Hsiung | 2010 | 0.43010145 | 0.36021858 | 0.49998432 |
| Hu | 2011 | 0.44675791 | 0.36654499 | 0.52697086 |
| Ito | 2012 | 0.45302898 | 0.37845969 | 0.52759826 |
| Jaworowska | 2011 | 0.45183578 | 0.37681371 | 0.52685785 |
| Jin | 2009 | 0.4566333 | 0.38218915 | 0.53107744 |
| Kohno | 2011 | 0.45396003 | 0.38049594 | 0.52742416 |
| Lan | 2013 | 0.44141158 | 0.36871165 | 0.51411152 |
| Li | 2016 | 0.4495545 | 0.37521073 | 0.5238983 |
| Liu | 2015 | 0.44411442 | 0.37005466 | 0.51817417 |
| Mandour | 2020 | 0.44980368 | 0.37680554 | 0.52280182 |
| Miki | 2010 | 0.44534221 | 0.36808807 | 0.52259636 |
| Myneni | 2013 | 0.44918022 | 0.37463668 | 0.52372372 |
| Shiraishi | 2016 | 0.44246551 | 0.36354977 | 0.52138126 |
| Shiraishi | 2012 | 0.43832248 | 0.36281666 | 0.51382834 |
| Truong(Asian) | 2010 | 0.45174417 | 0.37585378 | 0.52763456 |
| Truong(Caucasian) | 2010 | 0.46194831 | 0.39170203 | 0.53219461 |
| Wang | 2014 | 0.45088503 | 0.37507495 | 0.52669513 |
| Wang | 2016 | 0.44256231 | 0.36831301 | 0.51681161 |
| Wang | 2010 | 0.44615829 | 0.37179253 | 0.52052402 |
| Wei | 2015 | 0.44769791 | 0.37210333 | 0.52329248 |
| Xing | 2016 | 0.44138175 | 0.36851308 | 0.5142504 |
| Yin | 2014 | 0.44378048 | 0.3693327 | 0.51822829 |
| Yoo | 2020 | 0.45576903 | 0.38198888 | 0.52954912 |
| Yoon | 2010 | 0.4466922 | 0.37042198 | 0.52296239 |
| Combined |  | 0.44773976 | 0.37466533 | 0.5208142 |

**Table S4 Sensitivity analysis results of CA vs.AA**

| Study omitted | Year | Estimate | [95% Conf. Interval] | |
| --- | --- | --- | --- | --- |
| Bae | 2012 | 0.23098794 | 0.18342564 | 0.27855024 |
| Chen | 2012 | 0.22066474 | 0.17173637 | 0.26959309 |
| Dong | 2017 | 0.21828443 | 0.17013966 | 0.26642922 |
| Furuie | 2021 | 0.22602072 | 0.17755087 | 0.27449059 |
| Hosgood | 2015 | 0.21182016 | 0.16511421 | 0.25852609 |
| Hsiung | 2010 | 0.21240593 | 0.164391 | 0.26042086 |
| Hu | 2011 | 0.21858847 | 0.16535516 | 0.2718218 |
| Ito | 2012 | 0.22401755 | 0.17455119 | 0.2734839 |
| Jaworowska | 2011 | 0.22897033 | 0.18092468 | 0.27701601 |
| Jin | 2009 | 0.22289698 | 0.1728546 | 0.27293935 |
| Kohno | 2011 | 0.22885372 | 0.18173869 | 0.27596873 |
| Lan | 2013 | 0.21884838 | 0.17053582 | 0.26716092 |
| Li | 2016 | 0.22018822 | 0.17105703 | 0.26931939 |
| Liu | 2015 | 0.22440615 | 0.17574245 | 0.27306986 |
| Mandour | 2020 | 0.22352932 | 0.17605333 | 0.27100533 |
| Miki | 2010 | 0.21810874 | 0.16695166 | 0.26926583 |
| Myneni | 2013 | 0.22893494 | 0.18190777 | 0.27596211 |
| Shiraishi | 2016 | 0.21643494 | 0.16391438 | 0.2689555 |
| Shiraishi | 2012 | 0.21796958 | 0.16588642 | 0.27005276 |
| Truong(Asian) | 2010 | 0.22400762 | 0.17379256 | 0.2742227 |
| Truong(Caucasian) | 2010 | 0.22589105 | 0.1748917 | 0.27689043 |
| Wang | 2014 | 0.22429979 | 0.17427909 | 0.27432048 |
| Wang | 2016 | 0.21860681 | 0.16955937 | 0.26765427 |
| Wang | 2010 | 0.21999297 | 0.17109664 | 0.26888928 |
| Wei | 2015 | 0.22340232 | 0.17364217 | 0.27316245 |
| Xing | 2016 | 0.21652912 | 0.16840607 | 0.26465216 |
| Yin | 2014 | 0.21862967 | 0.16953644 | 0.26772287 |
| Yoo | 2020 | 0.22771628 | 0.17938463 | 0.27604795 |
| Yoon | 2010 | 0.2209314 | 0.17043874 | 0.27142406 |
| Combined |  | 0.22183554 | 0.17353801 | 0.27013307 |

**Table S5 Sensitivity analysis results of CA+CC vs.AA**

| Study omitted | Year | Estimate | [95% Conf. Interval] | |
| --- | --- | --- | --- | --- |
| Bae | 2012 | 0.28982204 | 0.23816951 | 0.34147459 |
| Chen | 2012 | 0.27925217 | 0.22658662 | 0.3319177 |
| Dong | 2017 | 0.27672231 | 0.22451842 | 0.32892621 |
| Furuie | 2021 | 0.28879496 | 0.23760061 | 0.33998933 |
| Hosgood | 2015 | 0.26875877 | 0.2184151 | 0.31910247 |
| Hsiung | 2010 | 0.26917097 | 0.21839023 | 0.31995174 |
| Hu | 2011 | 0.27813342 | 0.22080344 | 0.3354634 |
| Ito | 2012 | 0.28372642 | 0.23058558 | 0.33686727 |
| Jaworowska | 2011 | 0.28703704 | 0.23458128 | 0.3394928 |
| Jin | 2009 | 0.28350911 | 0.22995664 | 0.33706155 |
| Kohno | 2011 | 0.28863013 | 0.23761185 | 0.3396484 |
| Lan | 2013 | 0.27633235 | 0.22441684 | 0.32824787 |
| Li | 2016 | 0.27952284 | 0.22658023 | 0.33246544 |
| Liu | 2015 | 0.28160265 | 0.22882266 | 0.33438265 |
| Mandour | 2020 | 0.2819418 | 0.2302424 | 0.33364123 |
| Miki | 2010 | 0.27722871 | 0.22240593 | 0.33205149 |
| Myneni | 2013 | 0.28642011 | 0.23443307 | 0.33840713 |
| Shiraishi | 2016 | 0.27556705 | 0.21948981 | 0.33164433 |
| Shiraishi | 2012 | 0.2753053 | 0.21977229 | 0.33083829 |
| Truong(Asian) | 2010 | 0.2831687 | 0.22920334 | 0.33713406 |
| Truong(Caucasian) | 2010 | 0.28536841 | 0.23115604 | 0.3395808 |
| Wang | 2014 | 0.28305 | 0.22918332 | 0.33691671 |
| Wang | 2016 | 0.27631387 | 0.22353883 | 0.3290889 |
| Wang | 2010 | 0.27824879 | 0.22559714 | 0.33090043 |
| Wei | 2015 | 0.28129044 | 0.22765321 | 0.33492771 |
| Xing | 2016 | 0.27395752 | 0.22199138 | 0.32592365 |
| Yin | 2014 | 0.27676427 | 0.22384705 | 0.32968152 |
| Yoo | 2020 | 0.28741834 | 0.23534136 | 0.3394953 |
| Yoon | 2010 | 0.27988729 | 0.22543223 | 0.33434233 |
| Combined |  | 0.28047568 | 0.22846316 | 0.33248819 |

**Table S6 Sensitivity analysis results of CC vs.AA+CA**

| Study omitted | Year | Estimate | [95% Conf. Interval] | |
| --- | --- | --- | --- | --- |
| Bae | 2012 | 0.31872773 | 0.2610217 | 0.37643379 |
| Chen | 2012 | 0.31698361 | 0.26010782 | 0.37385944 |
| Dong | 2017 | 0.31706932 | 0.26025793 | 0.37388071 |
| Furuie | 2021 | 0.32538152 | 0.27132052 | 0.37944251 |
| Hosgood | 2015 | 0.30839202 | 0.25180796 | 0.36497611 |
| Hsiung | 2010 | 0.3034763 | 0.24919766 | 0.35775495 |
| Hu | 2011 | 0.31731334 | 0.25594601 | 0.37868068 |
| Ito | 2012 | 0.31924996 | 0.26198924 | 0.37651068 |
| Jaworowska | 2011 | 0.31569269 | 0.25796545 | 0.37341994 |
| Jin | 2009 | 0.32388788 | 0.26703745 | 0.38073829 |
| Kohno | 2011 | 0.31800652 | 0.26121098 | 0.37480202 |
| Lan | 2013 | 0.31295142 | 0.25685778 | 0.36904502 |
| Li | 2016 | 0.31903282 | 0.26215541 | 0.37591019 |
| Liu | 2015 | 0.31125188 | 0.25525063 | 0.36725312 |
| Mandour | 2020 | 0.31583858 | 0.25928539 | 0.37239176 |
| Miki | 2010 | 0.31623495 | 0.25672057 | 0.37574935 |
| Myneni | 2013 | 0.3129077 | 0.25610557 | 0.36970979 |
| Shiraishi | 2016 | 0.31349999 | 0.25265881 | 0.37434116 |
| Shiraishi | 2012 | 0.3068004 | 0.25077674 | 0.36282408 |
| Truong(Asian) | 2010 | 0.31889436 | 0.26052088 | 0.37726784 |
| Truong(Caucasian) | 2010 | 0.33426493 | 0.28683758 | 0.38169229 |
| Wang | 2014 | 0.3179695 | 0.25973901 | 0.37619999 |
| Wang | 2016 | 0.31382325 | 0.25678158 | 0.37086496 |
| Wang | 2010 | 0.31718251 | 0.25998792 | 0.3743771 |
| Wei | 2015 | 0.31531629 | 0.25735679 | 0.37327582 |
| Xing | 2016 | 0.31232649 | 0.25664902 | 0.36800393 |
| Yin | 2014 | 0.31496161 | 0.25771004 | 0.37221318 |
| Yoo | 2020 | 0.31992117 | 0.26292181 | 0.37692055 |
| Yoon | 2010 | 0.31547749 | 0.25700152 | 0.37395346 |
| Combined |  | 0.3161719 | 0.26013082 | 0.37221298 |

**Table S7 Sensitivity analysis results of NSCLC and SCLC**

| Study omitted | Year | Estimate | [95% Conf. Interval] | |
| --- | --- | --- | --- | --- |
| Chen NSCLC | 2012 | 0.19971311 | 0.15255904 | 0.24686716 |
| Chen SCLC | 2012 | 0.20254499 | 0.15582232 | 0.24926765 |
| Dong NSCLC | 2017 | 0.1992486 | 0.15203018 | 0.24646699 |
| Hsiung NSCLC | 2010 | 0.19042934 | 0.14475939 | 0.2360993 |
| Hu NSCLC | 2011 | 0.19929837 | 0.14866003 | 0.2499367 |
| Hu SCLC | 2011 | 0.20559761 | 0.15762901 | 0.25356621 |
| Jin NSCLC | 2009 | 0.20499286 | 0.15699202 | 0.2529937 |
| Kohno NSCLC | 2011 | 0.20680749 | 0.15995072 | 0.25366426 |
| Landi NSCLC | 2009 | 0.20464896 | 0.15721855 | 0.25207937 |
| Landi SCLC | 2009 | 0.21169935 | 0.16616407 | 0.25723463 |
| Mandour NSCLC | 2020 | 0.20193496 | 0.1551214 | 0.24874851 |
| Mandour SCLC | 2020 | 0.20117977 | 0.15455814 | 0.24780139 |
| Miki NSCLC | 2010 | 0.19888608 | 0.1497561 | 0.24801606 |
| Seow NSCLC | 2017 | 0.20663767 | 0.15898806 | 0.25428727 |
| Shiraishi NSCLC | 2016 | 0.19776605 | 0.14704239 | 0.24848971 |
| Shiraishi NSCLC | 2012 | 0.19563596 | 0.14697929 | 0.24429262 |
| Truong(Asian) NSCLC | 2010 | 0.20343824 | 0.15613212 | 0.25074437 |
| Truong(Asian) SCLC | 2010 | 0.20528358 | 0.15840812 | 0.25215903 |
| Truong(Caucasian) NSCLC | 2010 | 0.20324078 | 0.15505648 | 0.25142509 |
| Truong(Caucasian) SCLC | 2010 | 0.21302696 | 0.16905093 | 0.25700298 |
| Wang NSCLC | 2014 | 0.20254265 | 0.1539782 | 0.2511071 |
| Wang SCLC | 2010 | 0.20428017 | 0.15767436 | 0.25088596 |
| Wang NSCLC | 2010 | 0.19803368 | 0.15078622 | 0.24528112 |
| Wei NSCLC | 2015 | 0.20086208 | 0.1525995 | 0.24912466 |
| Xing NSCLC | 2016 | 0.19365208 | 0.14730273 | 0.24000144 |
| Yin NSCLC | 2014 | 0.19493167 | 0.14796741 | 0.24189593 |
| Yoon NSCLC | 2010 | 0.19991486 | 0.15119591 | 0.2486338 |
| Zhao NSCLC | 2013 | 0.19591075 | 0.14943394 | 0.24238755 |
| Combined |  | 0.20147317 | 0.15491978 | 0.24802656 |

**Table S8 Sensitivity analysis results of LUAD and LUSC**

| Study omitted | Year | Estimate | [95% Conf. Interval] | |
| --- | --- | --- | --- | --- |
| Chen LUAD | 2012 | 0.20174257 | 0.15575004 | 0.24773511 |
| Chen LUSC | 2012 | 0.21048442 | 0.1649833 | 0.25598553 |
| Hsiung LUAD | 2010 | 0.19506486 | 0.15028338 | 0.23984635 |
| Hsiung LUSC | 2010 | 0.20684375 | 0.1601688 | 0.2535187 |
| Hu LUSC | 2011 | 0.20854968 | 0.16003324 | 0.2570661 |
| Hu LUAD | 2011 | 0.20092379 | 0.1531948 | 0.24865277 |
| Jin LUAD | 2009 | 0.20242585 | 0.15585262 | 0.24899909 |
| Jin LUSC | 2009 | 0.20985688 | 0.16362117 | 0.25609261 |
| Kohno LUSC | 2011 | 0.21091022 | 0.16470441 | 0.25711602 |
| Landi LUAD | 2009 | 0.20575114 | 0.15787567 | 0.25362659 |
| Landi LUSC | 2009 | 0.21592601 | 0.17138398 | 0.26046804 |
| Mandour LUAD | 2020 | 0.2051347 | 0.15909597 | 0.25117344 |
| Mandour LUSC | 2020 | 0.20683093 | 0.16106974 | 0.25259212 |
| Miki LUAD | 2010 | 0.20365068 | 0.15559031 | 0.25171107 |
| Seow LUAD | 2017 | 0.21062215 | 0.1633102 | 0.25793409 |
| Shiraishi LUAD | 2016 | 0.20262489 | 0.15316926 | 0.2520805 |
| Shiraishi LUAD | 2012 | 0.20082422 | 0.15304855 | 0.24859989 |
| Truong(Asian) LUAD | 2010 | 0.20257919 | 0.15532629 | 0.24983206 |
| Truong(Asian) LUSC | 2010 | 0.21463315 | 0.1691144 | 0.26015189 |
| Truong(Caucasian) LUAD | 2010 | 0.20711093 | 0.15854798 | 0.25567389 |
| Truong(Caucasian) LUSC | 2010 | 0.21384045 | 0.16840079 | 0.25928012 |
| Wang LUAD | 2014 | 0.20304713 | 0.15584022 | 0.25025403 |
| Wang LUSC | 2014 | 0.20952991 | 0.16260453 | 0.25645527 |
| Wang LUAD | 2010 | 0.20259181 | 0.15633339 | 0.24885021 |
| Wang LUSC | 2010 | 0.20487867 | 0.15865602 | 0.25110134 |
| Yin LUAD | 2014 | 0.19985448 | 0.15357371 | 0.24613525 |
| Yoon LUAD | 2010 | 0.20171624 | 0.15448175 | 0.24895073 |
| Yoon LUSC | 2010 | 0.21030453 | 0.16367342 | 0.25693563 |
| Zhao LUAD | 2013 | 0.20050684 | 0.15496854 | 0.24604513 |
| Zhao LUSC | 2013 | 0.20528576 | 0.15911865 | 0.25145283 |
| Combined |  | 0.2057927 | 0.15993031 | 0.25165509 |
